# CD163⁺ monocytes and soluble CD163 as prognostic indicators in severe fever with thrombocytopenia syndrome: An integrative analysis

**DOI:** 10.1371/journal.pntd.0014416

**Published:** 2026-06-03

**Authors:** Jie Wang, Haolin Song, Yi Zhang, Ziling Cheng, Lingtong Huang, Bei Jia, Guangqi Zhu, Lifen Hu, Jihua Xue, Jie Li, Wei Wu, Qi Xia

**Affiliations:** 1 State Key Laboratory for Diagnosis and Treatment of Infectious Diseases, National Clinical Research Center for Infectious Diseases, National Medical Center for Infectious Diseases, Collaborative Innovation Center for Diagnosis and Treatment of Infectious Diseases, The First Affiliated Hospital, Zhejiang University School of Medicine, Hangzhou, Zhejiang, China; 2 Department of Laboratory Medicine, the First Affiliated Hospital, Zhejiang University School of Medicine, Hangzhou, Zhejiang, China; 3 Department of Critical Care Units, the First Affiliated Hospital, Zhejiang University School of Medicine, Hangzhou, Zhejiang, China; 4 Department of Infectious Diseases, Nanjing Drum Tower Hospital, Affiliated Hospital of Medical School, Nanjing University, Nanjing, Jiangsu, China; 5 Department of Infectious Diseases, the First Affiliated Hospital of Anhui Medical University, Hefei, Anhui, China; Colorado State University, UNITED STATES OF AMERICA

## Abstract

**Background:**

Severe fever with thrombocytopenia syndrome (SFTS) carries high short-term mortality and lacks approved antiviral therapy, highlighting the need for admission risk tools grounded in upstream host immune pathobiology.

**Methods:**

Public single-cell RNA sequencing (scRNA-seq) data (GSE175499) from 15 SFTS patients (4 non-survivors, 11 survivors) and 4 healthy controls were reanalyzed to map cell–cell communication and identify monocyte subsets and pathways. Findings were validated by bulk RNA sequencing and flow cytometry. A multicenter clinical cohort (n = 150, 132 survivors and 18 non-survivors) measured admission sCD163 using enzyme-linked immunosorbent assay (ELISA) and assessed 30-day mortality using area under the curve (AUC), Kaplan–Meier, and Cox models.

**Results:**

Single-cell analysis identified expansion of CD163 ⁺ intermediate monocytes in SFTS, along with antiviral and complement activation programs in non-survivors. Communication analysis prioritized thrombospondin (THBS) signaling with the dominant sender shifting from CD163 ⁺ intermediate (survivors) to CD163 ⁺ classical monocytes (non-survivors). Flow cytometry confirmed increased CD163 ⁺ monocytes in SFTS. At admission, sCD163 independently predicted 30-day mortality (optimal threshold = 1.17 µg/mL, AUC 0.80). A two-marker model combining sCD163 with blood urea nitrogen (BUN) improved discrimination (AUC 0.87), yielded stepwise separation across three risk tiers (Score 0, 1, and 2) and replicated externally (AUC 0.73 and 0.83). Elevated sCD163 was consistently associated with higher mortality across sex and age subgroups. However, further subgroup analyses within the three-tier risk score were limited by small sample sizes and should be considered exploratory.

**Conclusions:**

We identified and orthogonally validated CD163 ⁺ monocyte programs linked to outcomes, establishing admission serum sCD163 as a biomarker. An admission two-marker score (sCD163 and BUN) provides a simple three-tier admission score that rapidly stratifies 30-day mortality risk and guides intensified monitoring and timely supportive care.

## Author summary

Severe fever with thrombocytopenia syndrome (SFTS) is an emerging tick-borne viral disease with high mortality and limited treatment options. Current prognostic markers predominantly capture downstream organ injury, and upstream immune indicators are needed. CD163 is a scavenger receptor on monocytes/macrophages, and its soluble form (sCD163) has been linked to disease severity and mortality in several inflammatory diseases. In this study, we integrated single-cell RNA sequencing, bulk transcriptomics, and flow cytometry to characterize CD163 ⁺ monocyte subsets in SFTS. A multicenter cohort validated admission sCD163, alone or combined with blood urea nitrogen (BUN), as a robust prognostic biomarker for 30-day mortality and as the basis for a simple three-tier risk stratification tool.

## Introduction

Severe fever with thrombocytopenia syndrome (SFTS) is an emerging tick-borne viral infection with substantial mortality in East Asia [1,2]. Contemporary cohorts report overall case-fatality ranging from approximately 7% to exceeding 20%, with higher risk in elderly patients [3]. In the absence of approved virus-specific therapy, clinical management remains largely supportive [4]. Early recognition of high-risk patients and prompt supportive management with close monitoring are considered critical in SFTS, although no virus-specific therapy has been approved and definitive mortality-reduction evidence remains limited. Accordingly, there is a pressing need for reliable admission-time risk stratification to identify patients at high risk of early death and to guide intensified monitoring and timely interventions.

Multiple clinical studies have shown that laboratory markers such as white blood cell (WBC) count, platelet (PLT) count, alanine aminotransferase (ALT), aspartate aminotransferase (AST), creatinine (Cr), activated partial thromboplastin time (APTT), D-dimer, and ferritin, either alone or in combination, are associated with disease severity and outcomes in SFTS patients [5–8]. Nevertheless, these serological indicators largely capture downstream tissue injury rather than the upstream cellular programs that drive fulminant disease, limiting their utility for early risk stratification. This gap motivates an approach that directly characterizes upstream inflammatory pathways at presentation.

Monocytes are central to SFTS immunopathogenesis and systemic inflammation [9]. CD163, a receptor expressed on alternatively activated monocytes, defines a subset of CD163 ⁺ monocytes associated with adverse outcomes in acute-on-chronic liver failure (ACLF). Its soluble form, sCD163, is an established indicator of inflammatory activation in severe infections such as sepsis, hemorrhagic fever with renal syndrome (HFRS), and HIV infection [10–13]. However, the molecular signaling programs underlying infection and disease severity, and their associations with clinical outcomes, remain insufficiently defined [14].

Here, we pursue a translational strategy spanning discovery to bedside. We integrated single-cell RNA sequencing (scRNA-seq) from public dataset (GSE175499) with multicenter clinical cohorts to: (1) define the transcriptional heterogeneity of CD163 monocyte subsets in surviving and non-surviving SFTS patients, and delineate signaling programs that underlie their functional differences; (2) orthogonally validate these signals using bulk transcriptomics and flow cytometry; (3) evaluate admission CD163 expression and serum sCD163 as prognostic indicators of 30-day all-cause mortality. This integrated approach aims to advance mechanistic understanding of SFTS immunopathogenesis and provide a clinically applicable tool for early patient risk stratification.

## Methods

### Ethics statement

The study was approved by the Ethics Committee of the First Affiliated Hospital, Zhejiang University School of Medicine (approval number: IIT20210312B-R1), the Ethics Committee of Nanjing Drum Tower Hospital (approval number: 2022-238-02), and the Ethics Committee of Anhui Medical University (approval number: PJ 2024-07-99). Written informed consent was obtained from all patients involved in this study.

### Study design

This was a multicenter, retrospective translational study comprising three components—scRNA-seq re-analysis, orthogonal validation, and a clinical cohort. Institutional review boards approved the protocol, and informed consent was obtained per local regulations. Reporting followed STROBE/REMARK guidelines.

### Single-cell RNA-seq analyses

Single-cell RNA-seq data (GSE175499) were processed with Cell Ranger v3.1.0 against the GRCh38 reference to generate gene–cell UMI matrices, which were imported into Seurat v5.3.0 for standard preprocessing (quality control, normalization, variable-feature selection, scaling, PCA, UMAP embedding, neighbor graph construction, and clustering). Low-quality cells were removed by retaining cells with 500–8,000 detected genes and ≤25% mitochondrial transcripts; filtered data were downsampled as needed and visualized in 2D with UMAP. Cell clusters were annotated using canonical markers, with marker genes identified by FindAllMarkers (default settings) and annotations cross-checked against the Annotation of Cell Types resource and manual curation. For monocyte-focused analysis, subclustering was performed in Seurat using 10 principal components and a clustering resolution of 0.2. Differentially expressed genes were analyzed for function using clusterProfiler: Gene Ontology (GO) enrichment with org.Hs.e.g.,db and Kyoto Encyclopedia of Genes and Genomes (KEGG) pathway enrichment (organism = “hsa”), both with p-value and q-value cutoffs of 0.05. Cell–cell communication was inferred with CellChat by integrating expression and cell-type labels to quantify ligand–receptor interactions and compare signaling strength between groups.

### Orthogonal validation

#### Flow cytometry.

Peripheral venous blood was collected within 24 hours of hospital admission into EDTA anticoagulant tubes. EDTA-anticoagulated whole blood from SFTS patients and healthy controls was stained for 30 min at 4 °C in the dark with a pre-optimized antibody cocktail: anti-CD14-FITC, anti-CD16-BV510, anti-CD163-BV421, and anti- human leukocyte antigen DR (HLA-DR)-APC (BioLegend, USA). Red blood cells were lysed with BD FACS Lysing Solution (BD Biosciences) for 15 min at room temperature in the dark, followed by two washes with cold PBS containing 1% BSA. Samples were acquired on a BD FACSCanto II flow cytometer (BD Biosciences, San Jose, CA, USA).

#### Bulk RNA sequencing.

Total RNA was extracted from peripheral blood mononuclear cells (PBMCs) using TRIzol reagent (Thermo Fisher, 15596018) according to the manufacturer’s instructions. RNA quantity and purity were assessed with an Agilent 2100 Bioanalyzer and RNA 6000 Nano Kit (Agilent, USA). Samples with RIN > 7.0 were used for library preparation and sequenced on an Illumina NovaSeq 6000 platform (LC-Bio Technologies, Hangzhou, China).

### Clinical cohort (multi-center retrospective)

#### Setting and patients, baseline sampling.

We conducted a retrospective, multicenter cohort study of consecutive adults with laboratory-confirmed SFTS. The discovery cohort comprised patients admitted to Nanjing Drum Tower Hospital and the First Affiliated Hospital of Zhejiang University School of Medicine between May 2017 and November 2024. The external validation cohort included patients from the First Affiliated Hospital of Anhui Medical University between March 1 and June 30, 2025.

Inclusion criteria. Eligibility required laboratory-confirmed SFTSV infection, operationalized as meeting ≥1 of the following criteria: (i) positive serum viral RNA; (ii) seroconversion or a ≥ 4-fold rise in antibody titer between paired sera collected >2 weeks apart; or (iii) virus isolation from cell culture.

Exclusion criteria. Patients were excluded if any of the following conditions were present: (i) patients positive for other tick-borne pathogens; (ii) lacking laboratory confirmation. Clinical data were collected from medical records.

#### Serum sCD163 measurement.

Serum samples were obtained within 24 hours of admission, prior to corticosteroid administration when applicable. Serum was isolated from whole blood by centrifugation at 3000 rpm (≈1500 × g) for 10 min, and the supernatant was collected for analysis. Soluble CD163 (sCD163) was quantified using a commercial ELISA kit (Human sCD163 ELISA Kit; Cat. No. EK1110; Hangzhou Multi Sciences Biotech Co., Ltd., China) strictly according to the manufacturer’s instructions; the assay has a detection range of 62.5–4000 pg/mL and a sensitivity of 4.39 pg/mL.

#### Outcomes and follow-up.

The primary endpoint was 30-day all-cause mortality from the date of hospital admission. Survival outcomes were recorded at discharge for most patients; for those unexpectedly discharged due to clinical deterioration, follow-up telephone calls were conducted to confirm vital status and the date of death. Survivors were censored at day 30.

#### Data collection and statistical analysis.

Data for analysis were extracted from medical records using a structured framework encompassing demographics, comorbidities, clinical manifestations and disease course, treatments, and laboratory results. The primary outcome was all-cause mortality. Follow-up time was calculated from the date of hospital admission to the date of death for non-survivors and to the end of follow-up for survivors. Continuous variables were summarized as median (interquartile range, IQR) owing to skewed distributions, and categorical variables as counts (percentages). Between-group comparisons (survivors vs non-survivors) were performed accordingly. Mortality hazard ratios (HRs) with 95% confidence intervals (CIs) were estimated using Cox proportional hazards models: demographic characteristics and laboratory parameters were first evaluated in univariable analyses, and variables with *P* < 0.05 were entered into multivariable models. Survival differences among groups stratified by sCD163 were compared using Kaplan–Meier methods with log-rank tests. Discriminative performance for mortality was assessed with receiver operating characteristic (ROC) curves for sCD163. All analyses were conducted in R (version 4.4.3), and statistical significance was defined as two-sided *P* < 0.05.

## Results

### Mechanistic discovery in single-cell datasets

#### CD163 ⁺ monocytes dynamics in SFTS patients.

**Fig 1:**
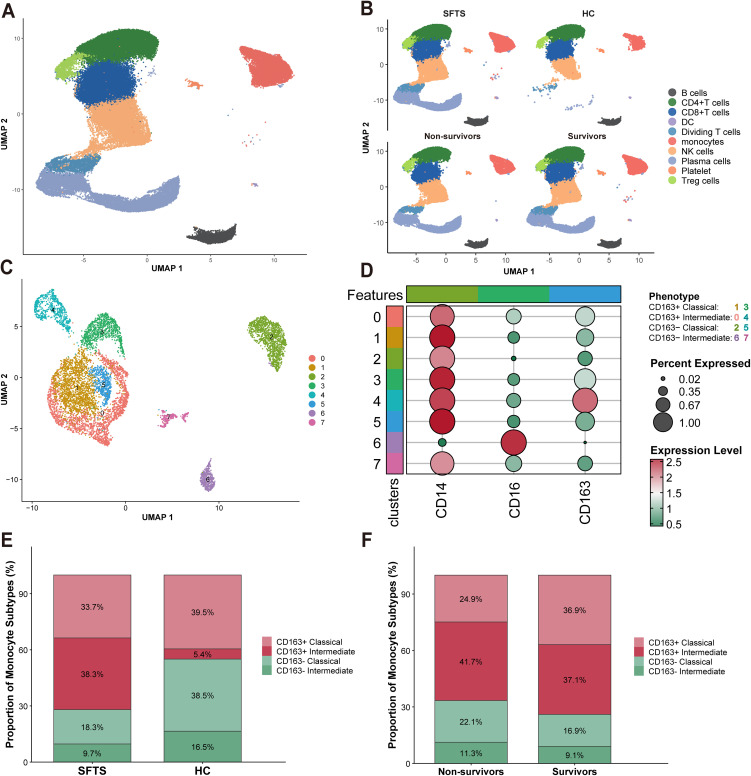
Single-cell transcriptomic profiling of PBMCs and monocyte subset composition in SFTS patients. **(A)** UMAP embedding of 95,739 PBMCs from 19 individuals (15 SFTS patients and 4 healthy controls), annotated into ten major immune cell types and colored by cell type. **(B)** UMAP plots stratified by cohort (healthy controls, SFTS overall, non-survivors, survivors), colored by immune cell type. **(C)** UMAP of monocytes after subclustering into eight clusters. **(D)** Dot plot showing CD14, CD16, and CD163 expression across monocyte clusters (dot size, percent expressing; color, scaled average expression). **(E)** Stacked bar charts comparing monocyte-subset proportions between healthy controls and SFTS patients. **(F)** Stacked bar charts comparing monocyte-subset proportions between non-survivors and survivors.

From previously published scRNA-seq datasets (GSE175499), we analyzed circulating PBMCs from 15 SFTS patients (11 survivors and 4 non-survivors) and 4 healthy individuals, comprising a total of 95,739 cells. Ten major immune cell types were identified with monocytes exhibiting significant compositional changes across healthy controls, survivors and non-survivors (Fig 1A–B). Monocyte subclusters were annotated based on their global transcriptomic profiles, guided by the expression of canonical markers and established monocyte classification frameworks in human peripheral blood (Fig 1C-D) [15]. Classical monocytes were characterized by dominant CD14 expression with low CD16, while intermediate monocytes exhibited relatively higher CD16 expression. Each subset was further stratified into CD163⁺ and CD163 ⁻ populations based on expression of the scavenger receptor CD163 (Fig 1C-D). Collectively, this annotation identified four main phenotypes from eight clusters: CD163 ⁺ classical (clusters 1 and 3), CD163 ⁺ intermediate (clusters 0 and 4), CD163 ⁻ classical (clusters 2 and 5), and CD163 ⁻ intermediate (clusters 6 and 7). Compared with healthy controls, SFTS patients exhibited a significant expansion of total CD163 ⁺ monocytes (72.0% vs. 44.8%, *P* = 0.024), especially CD163 ⁺ intermediate monocytes (38.3% vs. 5.4%, *P* = 0.003, Fig 1E). Compared with survivors, non-survivors showed a numerically higher proportion of CD163 ⁺ intermediate monocytes (41.7% vs. 37.1%) and a lower proportion of CD163 ⁺ classical monocytes (24.9% vs. 36.9%), although these differences were not statistically significant (Fig. 1F).

#### CD163 ⁺ monocytes exhibit an enhanced antiviral and complement transcriptional program in non-survivors.

**Fig 2:**
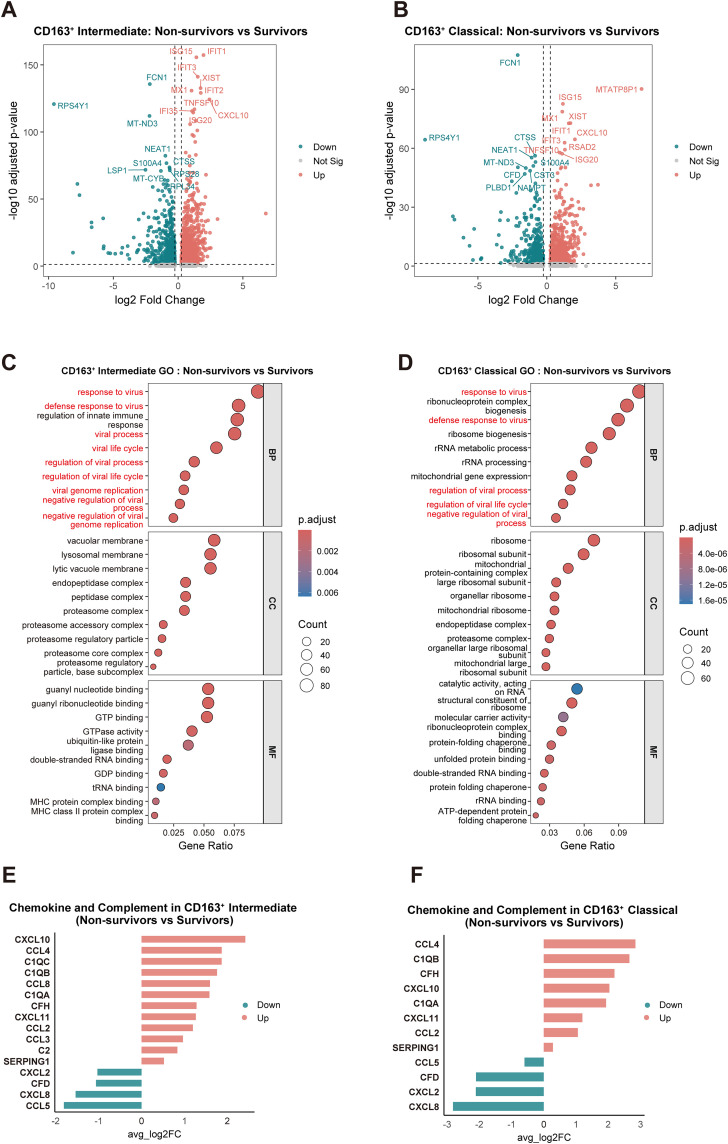
Differential gene expression and functional enrichment in CD163 ^+^ monocyte subsets comparing non-survivors and survivors. **(A–B)** Volcano plots showing differentially expressed genes (DEGs) between non-survivors and survivors in CD163 ⁺ intermediate (A) and CD163 ⁺ classical (B) monocytes. Red and blue dots indicate genes significantly up- or down-regulated (adjusted *P* < 0.05, |log₂FC| > 0.25). **(C–D)** Gene Ontology (GO) enrichment analyses of DEGs in CD163 ⁺ intermediate (C) and CD163 ⁺ classical (D) monocytes. Dot size represents gene count, and color indicates adjusted *P* value. **(E–F)** Differential expression of chemokine- and complement-related genes between non-survivors and survivors in CD163 ⁺ intermediate (E) and CD163 ⁺ classical (F) monocytes (x-axis, log₂ fold change).

Differential gene expression (DEG) analyses between non-survivors and survivors revealed extensive transcriptional reprogramming in CD163 ⁺ monocytes. A total of 1,217 genes (811 upregulated and 406 downregulated) were significantly altered in CD163 ⁺ intermediate monocytes when comparing non-survivors with survivors, while 893 genes (605 upregulated and 288 downregulated) were differentially expressed in CD163 ⁺ classical monocytes (Fig 2A–B, S1–S2 Table).

GO enrichment analysis highlighted a robust antiviral transcriptional program in both CD163 ⁺ intermediate and classical subsets comparing non-survivors to survivors, with “response to virus” ranking as the top biological process (9/10 top enriched terms in CD163 ⁺ intermediate monocytes and 5/10 in the classical) (Fig 2C–D, S3–S4 Table). Functionally, both CD163 ⁺ intermediate and classical monocytes showed increased expression of IFN-inducible chemokines (CXCL10, CXCL11) and evidence of complement activation, with marked upregulation of classical-pathway components C1QA/C1QB and regulators such as SERPING1 (Fig 2E–F).

Within each monocyte compartment (classical and intermediate), GO enrichment analysis comparing CD163⁺ with their CD163 ⁻ counterparts revealed that CD163 ⁺ intermediate monocytes were significantly enriched in functional pathways related to oxidative stress, phagocytosis and viral process, while CD163 ⁺ classical monocytes were predominantly enriched in ribosome biogenesis, mitochondrial organization, rRNA processing, and ATP hydrolysis activity (S1 Fig, S5–S6 Table).

#### Cell–cell communication analysis identifies CD163 ⁺ monocytes as dominant signal senders and reveals attenuated THBS signaling in non-survivors.

**Fig 3:**
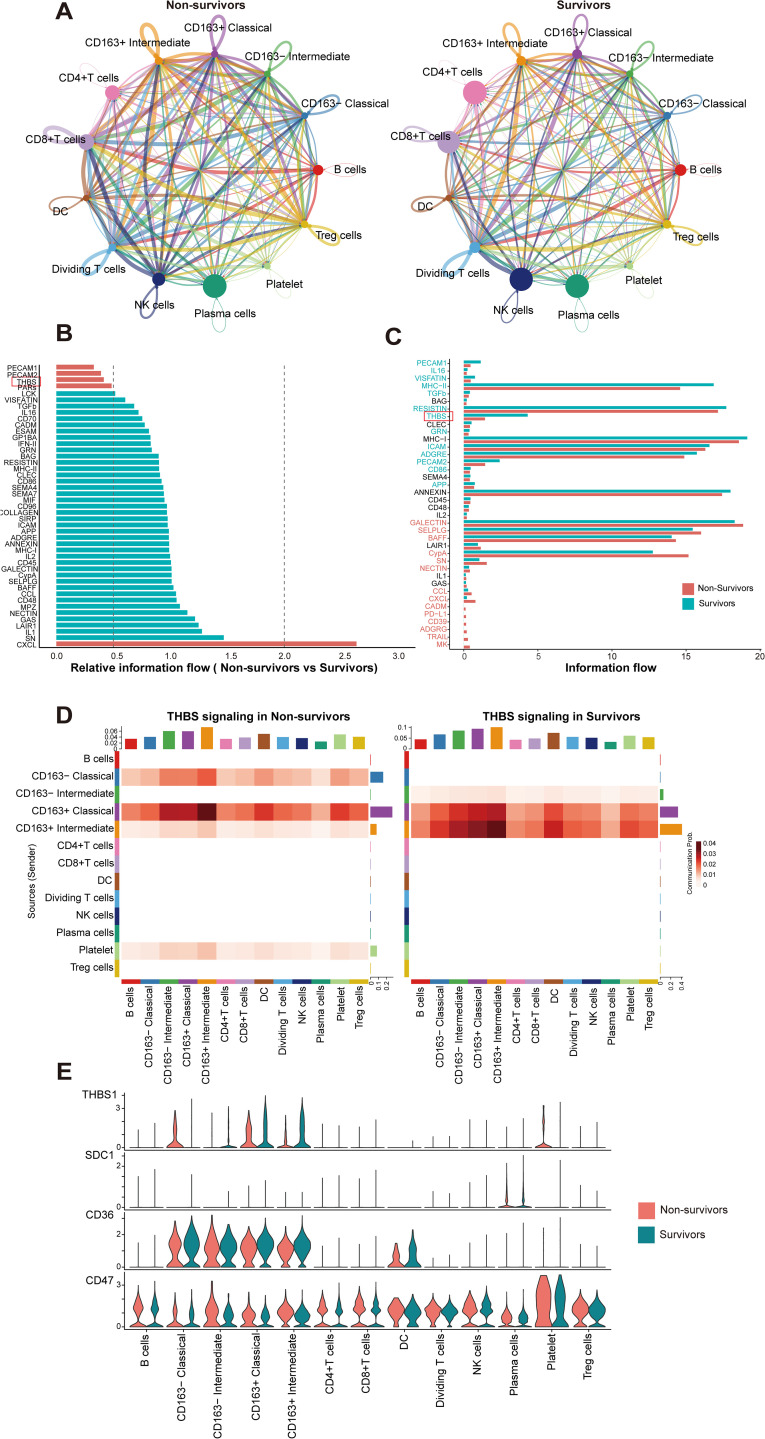
Intercellular interaction alterations between non-survivors and survivors in SFTSV patients. **(A)** Chord diagrams of inferred cell–cell communication networks in non-survivors (left) and survivors (right). Each line represents a predicted ligand–receptor interaction, with line thickness proportional to interaction strength. **(B)** Relative information flow (non-survivors vs. survivors) for each signaling pathway, red bars denote pathways showing ≥2-fold increase or decrease. **(C)** Total information flow (aggregated communication probability) of each signaling pathway in the two cohorts (red = non-survivors; blue = survivors). **(D)** THBS signaling heatmaps showing pairwise interaction strength between sending (rows) and receiving (columns) cell types in non-survivors (left) and survivors (right). **(E)** Violin plots of THBS1 and its receptors (SDC1, CD36, CD47) across major immune cell types, stratified by cohort (red = non-survivors; blue = survivors).

We performed cell–cell communication analysis across all signaling pathways to profile global sender-receiver patterns in non-survivors and survivors. Monocytes were the predominant sender cells in both groups, with the CD163 ⁺ intermediate monocytes showing the highest net sending strength. As to the receivers, CD163 ⁺ intermediate monocytes were also among the top in both groups, whereas CD8 ⁺ T cells showed the highest overall incoming signaling strength in non-survivors. Notably, CD163 ⁺ monocytes displayed strong outgoing communication toward CD8 ⁺ T cells in non-survivors (Fig 3A). Using a two-step ranking (relative strength followed by absolute aggregate signaling strength), we focused on the THBS pathway as it exhibited the largest absolute aggregate information flow among the top differentially strengthened pathways (Fig 3B–C). THBS pathway strength was lower in the non-survivor group than in the survivor group (Fig 3D). Across all cell types involved in THBS signaling, CD163 ⁺ monocytes were the principal senders in both groups with an outcome-associated shift from CD163 ⁺ intermediate monocytes as the dominant senders in survivors to CD163 ⁺ classical monocytes in non-survivors. At the receptor gene level, CD36 was preferentially expressed on CD163 ⁺ monocytes and was lower in non-survivors than in survivors, while SDC1 and CD47 did not display a consistent group-wise shift (Fig 3E).

### Bulk transcriptomics and flow cytometry orthogonally validate CD163 ⁺ monocyte expansion and functional dysregulation

**Fig 4:**
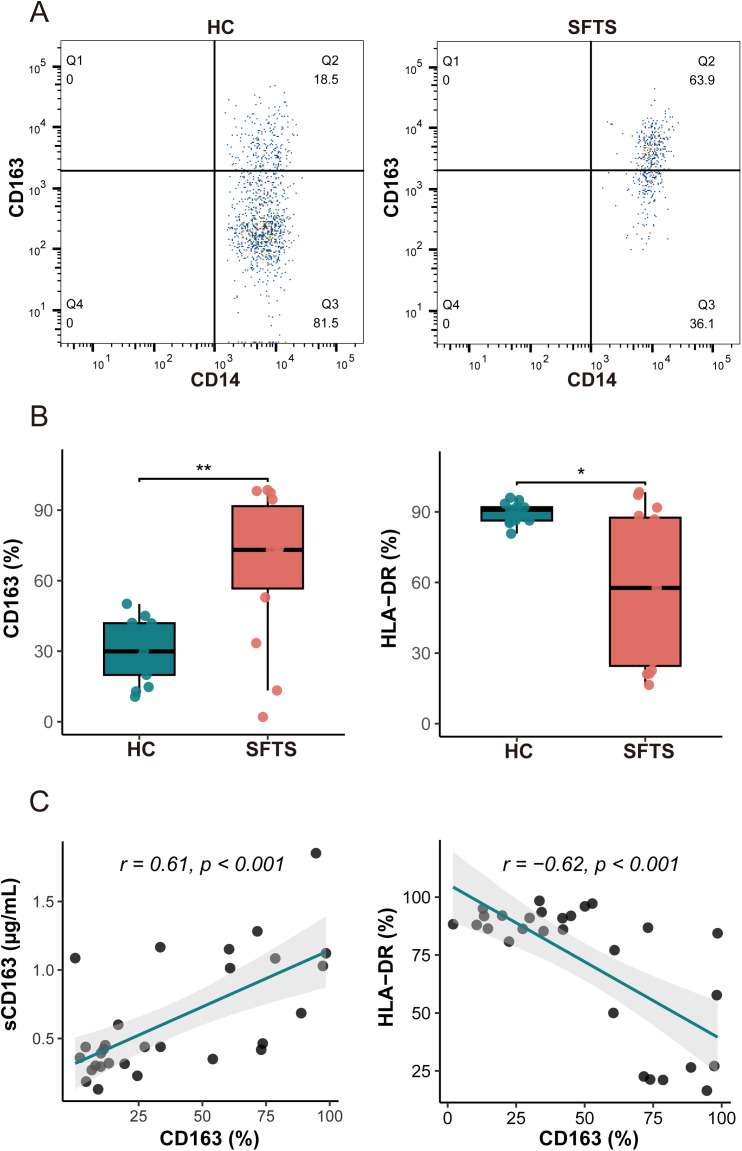
Flow cytometric profiling of monocyte markers and their correlations in SFTS patients. **(A)** Representative flow cytometry plots showing CD14 and CD163 expression on monocytes from a healthy control (HC) and an SFTS patient. **(B)** Flow cytometric quantification of CD163⁺ and HLA-DR⁺ monocyte frequencies in SFTS patients and HCs (*P < 0.05, **P < 0.01). **(C)** Correlation analyses showing positive association between serum sCD163 levels and CD163 ⁺ monocyte frequencies, and negative association between CD163⁺ and HLA-DR⁺ monocyte frequencies.

Transcriptomic analysis showed that CD163 expression was significantly elevated in patients with SFTS compared with healthy controls (HCs) (S2 Fig). Flow cytometric analysis revealed a marked increase in the proportion of CD163 ⁺ monocytes (CD14 ⁺ CD163⁺) in SFTS patients compared with healthy controls (HCs) (Fig 4A–B). In contrast, the frequency of HLA-DR⁺ monocytes, indicative of antigen-presenting capacity and immune activation, was significantly reduced in SFTS patients (Fig 4B). Correlation analysis demonstrated a strong negative association between the frequency of CD163 ⁺ monocytes and HLA-DR expression (r = –0.62, *P* < 0.001), and a positive correlation between CD163 ⁺ monocyte frequency and serum sCD163 concentrations (r = 0.61, *P* < 0.001) (Fig 4C).

### Clinical translation: biomarker discovery and validation

#### Demographic and clinical characteristics of the multicenter SFTS cohort.

We enrolled a discovery cohort consisting of 150 patients with SFTS from two medical centers during May 2017 to Nov 2024 (132 survivors and 18 non-survivors). The proportions of males, hypertension, type 2 diabetes, and cancer were comparable between groups. Median age was higher in non-survivors than in survivors (71.00 vs 62.00 years; *P* = 0.022). There were no statistically significant differences in the frequencies of headache (11.11% vs 18.18%, *P* = 0.740), bleeding tendency (27.78% vs 12.12%, *P* = 0.138), gastrointestinal symptoms (44.44% vs 54.55%, *P* = 0.459), or central nervous system (CNS) symptoms (16.67% vs 6.06%, *P* = 0.129). At admission, non-survivors had lower platelet counts and serum albumin (ALB) and more deranged renal and coagulation parameters than survivors: PLT 42.00 vs 67.00 × 10^9^/L (*P* = 0.013); ALB 29.50 vs 35.40 g/L (*P* = 0.004); ALT 100.00 vs 62.00 U/L (*P* = 0.039); AST 426.00 vs 116.00 U/L (*P* < 0.001); lactate dehydrogenase (LDH) 1819.00 vs 529.50 U/L (*P* < 0.001); creatinine 94.00 vs 66.65 µmol/L (*P* < 0.001); BUN 8.82 vs 5.15 mmol/L (*P* < 0.001); APTT 57.20 vs 37.30 s (*P* < 0.001). Serum level of sCD163 was higher (1.37 µg/mL vs 0.69 µg/mL, *P* < 0.001) in non-survivors versus survivors. Use of corticosteroids and intravenous immunoglobulin was more frequent in non-survivors (Table 1).

**Table 1:** Baseline characteristics of SFTS patients.

Variables	Discovery			Validation		
	Survivors N = 132	Non-survivors N = 18	*P* value	Survivors N = 49	Non-survivors N = 8	*P* value
**Demographic feature**						
Age(yr)	62.00 (54.00, 71.00)	71.00 (59.00, 77.00)	0.022	62.00 (54.00, 74.00)	70.00 (63.50, 73.50)	0.265
Gender, Male (%)	52 (39.39%)	9 (50.00%)	0.448	25(51.02%)	4 (50.00%)	>0.999
Hypertension	36 (27.27%)	6 (33.33%)	0.584	13 (27.66%)	2 (28.57%)	>0.999
Type 2 diabetes	18 (13.64%)	2 (11.11%)	>0.999	3(6.82%)	2(33.33%)	0.103
Cancer	7 (5.30%)	2 (11.11%)	0.300	3 (6.82%)	0(0.00%)	>0.999
**Clinical signs and symptoms**						
Headache	24 (18.18%)	2 (11.11%)	0.740	4 (9.30%)	0 (0.00%)	>0.999
Bleeding tendency	16 (12.12%)	5 (27.78%)	0.138	2 (4.76%)	2 (33.33%)	0.071
Gastrointestinal symptoms	72 (54.55%)	8 (44.44%)	0.459	27 (57.45%)	3 (42.86%)	0.687
CNS symptoms	8 (6.06%)	3 (16.67%)	0.129	3 (6.98%)	2 (28.57%)	0.130
**Laboratory tests**						
WBC(×10^9^/L)	3.00 (1.96, 4.94)	3.25 (1.90, 5.78)	0.688	3.59 (1.81, 5.77)	3.28 (1.66, 8.32)	0.842
PLT(×10^9^/L)	67.00 (42.50, 93.50)	42.00 (28.00, 70.00)	0.013	57.00 (44.00, 77.00)	44.50 (24.50, 50.00)	0.040
Hb(g/L)	130.00 (117.00, 141.00)	127.50 (110.00, 147.00)	0.923	131.00 (119.50, 142.50)	111.00 (101.50, 129.00)	0.037
ALT(U/L)	62.00 (37.80, 105.00)	100.00 (49.00, 260.00)	0.039	49.00 (33.00, 87.00)	49.50 (43.00, 146.50)	0.301
AST(U/L)	116.00 (58.00, 240.20)	426.00 (261.00, 935.00)	<0.001	84.00 (57.00, 158.00)	213.00 (83.50, 570.50)	0.058
LDH(U/L)	529.50 (298.00, 1,000.00)	1819.00 (941.00, 3,234.00)	<0.001	395.00 (294.00, 558.00)	918.50 (407.50, 2,740.50)	0.027
TB(µmol/L)	9.40 (6.40, 13.60)	13.65 (5.90, 19.60)	0.191	8.10 (6.50, 10.00)	9.30 (6.15, 13.50)	0.543
ALB(g/L)	35.40 (31.90, 39.10)	29.50 (27.20, 35.50)	0.004	36.00 (32.00, 38.00)	30.50 (28.75, 35.50)	0.115
GLB(g/L)	27.65 (23.90, 32.30)	31.45 (25.60, 38.20)	0.151	26.50 (24.70, 30.00)	29.30 (26.80, 31.85)	0.280
Cr(µmol/L)	66.65(54.85, 85.65)	94.00 (70.20, 139.00)	<0.001	75.00 (60.01, 86.37)	83.64 (71.72, 115.87)	0.132
BUN(mmol/L)	5.15 (3.70, 7.38)	8.82 (6.42, 11.96)	<0.001	5.97 (4.45, 7.68)	10.97 (6.67, 16.33)	0.004
PT(s)	11.50 (10.80, 12.20)	12.00 (10.50, 12.80)	0.356	12.10 (11.30, 12.80)	12.40 (11.85, 14.30)	0.232
APTT(s)	37.30 (32.30, 42.50)	57.20 (41.90, 69.60)	<0.001	52.50 (40.40, 61.60)	52.80 (46.80, 67.60)	0.445
sCD163(µg/mL)	0.69 (0.53, 0.97)	1.37 (1.19,2.23)	<0.001	0.82 (0.60, 1.03)	1.21 (0.84, 1.35)	0.042
**Treatment**						
Use of corticosteroid	30 (22.73%)	11 (61.11%)	0.001	22 (45.83%)	3 (42.86%)	>0.999
Use of ribavirin	121 (91.67%)	17 (94.44%)	>0.999	17 (36.17%)	4 (50.00%)	0.464
Use of immunoglobulin	48 (36.36%)	12 (66.67%)	0.020	33 (67.35%)	8 (100.00%)	0.090

#### Elevated sCD163 and BUN are independent predictors of 30-day mortality.

In univariable Cox analyses, older age, lower platelet counts and albumin, higher ALT, AST, LDH, Cr, BUN, and APTT, higher sCD163, and use of corticosteroids or immunoglobulin were each associated with increased mortality. After including variables significant in univariable analyses and excluding those with high multicollinearity (AST and Cr), the final multivariable Cox regression model identified higher sCD163 levels (HR 13.896, 95% CI 2.360–81.831, *P* = 0.004) and BUN (HR 1.172, 95% CI 1.004–1.368, *P* = 0.044) as independent predictors of mortality (Table 2). Consistent with these multivariable findings, ROC analysis demonstrated that the combination of sCD163 with BUN provided the highe st discriminative performance, forming the basis of the proposed risk score.

**Table 2:** The Cox regression analysis of mortality risk for the patients with severe fever with thrombocytopenia syndrome.

Variables	Univariate		Multivariate	
	HR (95%CI)	*P* value	HR (95%CI)	*P* value
Age(yrs)	1.045 (1.002 − 1.091)	0.042	1.025 (0.972 − 1.080)	0.360
Gender, Male(%)	1.501 (0.596 − 3.782)	0.389	a	
PLT(×109/L)	0.981 (0.965 − 0.998)	0.033	0.995 (0.972 − 1.018)	0.658
ALT(U/L)	1.004 (1.001 − 1.007)	0.012	0.993 (0.985 − 1.002)	0.296
AST(U/L)	1.002 (1.001 − 1.002)	<0.001	b	
LDH(U/L)	1.001 (1.000 − 1.001)	<0.001	1.000 (1.000 − 1.001)	0.391
ALB(g/L)	0.853 (0.765 − 0.952)	0.004	1.007 (0.983 − 1.031)	0.595
Cr(µmol/L)	1.004 (1.000 − 1.007)	0.036	b	
BUN(mmol/L)	1.145 (1.071 − 1.225)	<0.001	1.172 (1.004 − 1.368)	0.044
APTT(s)	1.011 (1.003 − 1.019)	0.005	1.022 (0.999 − 1.045)	0.056
High sCD163 group^c^	14.572 (4.742 − 44.774)	<0.001	13.896 (2.360 − 81.831)	0.004
Use of corticosteroid^c^	4.621 (1.791 − 11.927)	0.002	2.587 (0.378 − 17.717)	0.333
Use of immunoglobulin^c^	3.142 (1.179 − 8.373)	0.022	2.259 (0.370 − 13.808)	0.378

^a^Gender was not included in the multivariate model because its P value was > 0.05 in the univariate analysis. ^b^AST and Cr were not included in the multivariate Cox regression model due to multicollinearity. ^c^sCD163 was categorized into low group (≤ 1.17 µg/mL) and high group (> 1.17 µg/mL); “Use of corticosteroid” and “Use of immunoglobulin” were coded as binary variables (reference = No).

#### The three-tier risk score (sCD163 + BUN) provides stepwise stratification of 30-day mortality risk across discovery and validation cohorts.

**Fig 5:**
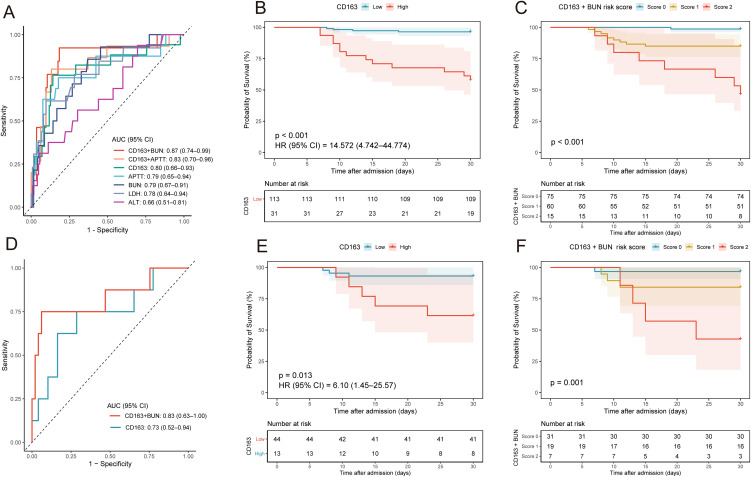
Prognostic value of sCD163 and the sCD163 + BUN three-tier score in SFTS discovery and validation cohorts. **Discovery cohort (A–C): (A)** ROC curves for 30-day mortality prediction comparing sCD163 with routine laboratory markers in SFTS patients. **(B)** Kaplan–Meier survival curves stratified by sCD163 levels using the optimal cutoff value of sCD163. **(C)** Kaplan–Meier curves based on a three-tier risk score combining sCD163 and BUN: Score 0, both normal (sCD163 ≤ 1.17 µg/mL and BUN ≤ 7.1 mmol/L); Score 1, one abnormal; Score 2, both abnormal. “Number at risk” is shown below. **External validation cohort (D–F)**: **(D)** ROC curves for mortality prediction comparing sCD163 and the combined sCD163 + BUN model. **(E)** Kaplan–Meier survival curves stratified by sCD163 levels using the same cutoff. **(F)** Kaplan–Meier curves based on the same three-tier risk score (Score 0–2) defined by sCD163 and BUN thresholds, confirming consistent risk stratification in the validation cohort.

ROC analysis demonstrated that sCD163 robustly discriminated 30-day mortality (AUC = 0.80, 95% CI 0.66–0.93), outperforming ALT (0.66, 0.51–0.81), comparable to APTT (0.79, 0.65–0.94), BUN (0.79, 0.67–0.91) and LDH (0.78, 0.64–0.94) (Fig 5A). The optimal cut-off was 1.17 µg/mL (sensitivity 0.77, specificity 0.86), stratifying patients into high (n = 31) and low sCD163 (n = 113) groups. The high sCD163 group exhibited significantly increased mortality (HR 14.572, 95% CI 4.74–44.77, *P* < 0.001) and significantly worse survival (*P* < 0.001, Fig 5B).

Given that BUN was independently associated with 30-day mortality (HR 1.172) and showed higher discriminative power, we combined sCD163 with BUN which further improved predictive accuracy (AUC 0.87, 0.74-0.99). Building on this, we derived a three-tier risk score (Score 0: both normal; Score 1: one abnormal; Score 2: both abnormal) using the cutoff value of sCD163 (1.17 µg/mL) and BUN (7.1 mmol/L). Patients were stratified into low-risk (Score 0, n = 75), mid-risk (Score 1, n = 60), and high-risk (Score 2, n = 15), which showed a clear stepwise separation of survival curves (*P* < 0.001, Fig 5C). Restricted cubic spline (RCS) analysis confirmed a linear dose-response relationship between continuous sCD163 and mortality risk indicating mortality risk increases linearly with sCD163 levels (overall *P* < 0.001, non-linearity *P* = 0.120, S3 Fig).

#### Subgroup and risk-stratified analyses.

**Fig 6:**
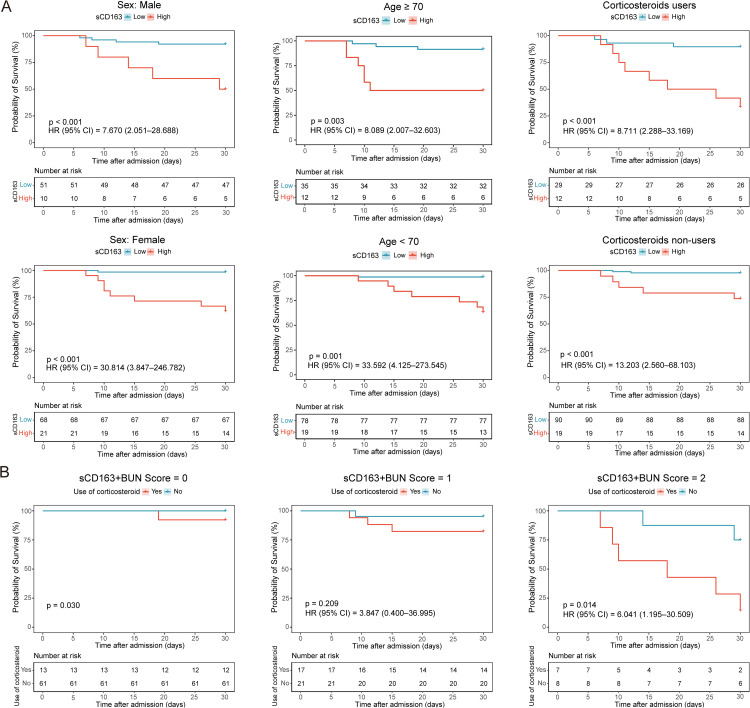
Risk stratification of subgroup analyses and steroid usage. **(A)** Kaplan–Meier curves comparing high vs low sCD163 within prespecified strata (male vs female, age ≥ 70 vs < 70 years, and corticosteroid users vs non-users). High sCD163 was defined as 1.17 µg/mL or higher. Numbers at risk are shown below each plot. Hazard ratios are estimated with Cox models; P values are from log-rank tests. **(B)** Kaplan–Meier curves comparing corticosteroid users and non-users within strata of the three-tier sCD163 + BUN score (0/1/2). One point for high sCD163 (1.17 µg/mL or higher) and one point for elevated BUN (7.1 mmol/L or higher). Log-rank P values (and, where shown, Cox hazard ratios) are displayed on the panels.

High sCD163 (≥ 1.17 µg/mL) consistently predicted higher mortality across prespecified subgroups (Fig 6). The association was evident in men (HR 7.670, 95% CI 2.051–28.688), women (HR 30.814, 95% CI 3.847–246.782), older patients aged ≥70 years (HR 8.090, 95% CI 2.012–32.600), younger patients aged <70 years (HR 33.590, 95% CI 4.130–273.550), patients receiving corticosteroids (HR 8.711, 95% CI 2.288–33.169), and those not receiving corticosteroids (HR 13.203, 95% CI 5.620–68.103) (all *P* < 0.01) (Fig 6A).

Using the three-tier CD163 + BUN score (0/1/2), exploratory analyses suggested heterogeneous survival differences by corticosteroid use (Fig 6B). In the high-risk stratum (Score 2), corticosteroid use was associated with higher mortality (HR 6.041, 95% CI 1.195–30.509; *P* = 0.014), whereas no significant association was observed in the intermediate stratum (Score 1) (HR 3.847, 95% CI 0.400–36.995; *P* = 0.209). Among low-risk patients (Score 0), survival curves separated modestly (*P* = 0.030). These observations should be interpreted with caution, as they may be influenced by confounders such as baseline disease severity. Similar trends were observed across age, sex, and corticosteroid use subgroups using the three-tier sCD163 + BUN risk score, but statistical reliability is limited by small sample sizes (S4 Fig).

#### External validation confirms the prognostic value of sCD163 and the combined risk score.

We enrolled 57 SFTS patients (49 survivors and 8 non-survivors) as external validation cohort from the First Affiliated Hospital of Anhui Medical University between March 1 and June 30, 2025. In the independent validation cohort, ROC analysis confirmed that sCD163 retained a strong discriminatory performance (AUC = 0.73, 95% CI 0.52–0.94), which was further improved when combined with BUN (AUC = 0.83, 95% CI 0.63–1.00) (Fig 5D). Using the same cutoffs for sCD163 (1.17 µg/mL) and BUN (7.1 mmol/L), patients were classified by sCD163 and the combined three-tier CD163 + BUN score. Consistent with the discovery cohort, elevated sCD163 and higher composite risk scores were both associated with significantly increased mortality and poorer short-term survival (*P* = 0.013 for sCD163; *P* = 0.001 for composite score) (Fig 5E–F).

## Discussion

In this study, we integrated single-cell profiling with multicenter clinical cohorts to link CD163 ⁺ monocyte dynamics in SFTS to an admission-time prognostic readout: serum sCD163, combined with BUN, enables a pragmatic three-tier risk score for 30-day mortality. By targeting upstream monocyte–macrophage activation rather than downstream injury, the approach supports early triage in emergency or fever clinics. Findings were reproduced in an external cohort, although thresholds may require prospective and risk-stratified validation.

Monocytes/macrophages orchestrate early antiviral defense and immunopathology in acute viral syndromes, positioning them earlier in the pathogenic cascade than tissue-injury readouts, with acute infection driving an influx of monocyte-derived macrophages that reshapes the macrophage compartment to promote pathogen clearance and restoration of homeostasis [16].

CD163 and its shed form (sCD163) mark alternatively activated monocytes/macrophages at the cellular level (membrane CD163) and in the circulation (soluble CD163) [14]. CD163 ⁺ monocytes are markedly increased in patients with progressive ACLF and correlate with adverse prognostic scores [10]. In SFTS, prior work has described a shift from classical to intermediate monocyte phenotypes, particularly among those with fatal outcomes [17]. Consistent with this intermediate-monocyte skewing, our single-cell analysis confirmed a significant expansion of CD163 ⁺ intermediate monocytes in SFTS patients compared with healthy controls. CD163 ⁺ intermediate monocytes were numerically more abundant in non-survivors than in survivors, but this difference was not statistically significant, likely owing in part to the limited number of non-survivors in the scRNA-seq cohort (n = 4). Nevertheless, CD163 ⁺ monocytes from non-survivors displayed distinct antiviral, inflammatory, and complement-related transcriptional programs. Concordantly, admission sCD163 levels were markedly elevated in non-survivors and remained independently associated with 30-day mortality after multivariable adjustment, supporting CD163 biology as a mechanistically grounded, admission-time prognostic axis.

Pathway-level analyses implicated CD163 ⁺ monocytes as an outcome-associated subset in non-survivors. This subset was characterized by upregulation of IFN-dependent chemokines (CXCL10) and complement genes (C1QA, C1QB), features consistent with amplified innate responses that can both control viruses and potentiate tissue injury. Ligand–receptor inference further identified CD163 ⁺ monocytes as the principal signal senders and CD8 ⁺ T cells as dominant signal receivers in non-survivors. Together with prior evidence that CXCL10–CXCR3 axes recruit CD8 ⁺ T cells from intermediate monocytes, these findings suggest an IFN-driven communication loop from CD163 ⁺ monocytes to CD8 ⁺ T cells that amplifies inflammatory responses in non-survivors [18,19].

THBS signaling ranked among the top differentially regulated pathways between outcome groups, with CD163 ⁺ monocytes as principal signal senders. Given that thrombospondin-1 (TSP1), encoded by THBS1, is typically low in healthy tissues but rapidly induced at sites of injury or infection, where it acts as a chemoattractant for neutrophils and monocytes/macrophages, the relative attenuation of THBS signaling observed in non-survivors (vs. survivors) raises the hypothesis that insufficient THBS-mediated resolution/clearance contributes to adverse outcomes [20–23].

Notably, CD163 ⁺ monocytes in SFTS exhibited a seemingly paradoxical phenotype, with increased expression of inflammatory and interferon-stimulated mediators but reduced HLA-DR surface expression. This pattern is consistent with sepsis-induced monocyte reprogramming, in which pro-inflammatory activation and immunosuppressive features can coexist during a dysregulated host response to infection [24,25]. Reduced monocyte HLA-DR expression is a hallmark of monocyte anergy or immunoparalysis and has been associated with impaired antigen presentation, secondary infections, and poor outcomes in sepsis [25]. Thus, CD163 ⁺ monocytes may represent a hyper-inflammatory yet immunoparalyzed state in severe SFTS, potentially amplifying tissue-damaging inflammation while impairing adaptive antiviral immune coordination.

Previous studies have highlighted several laboratory indicators reflecting parenchymal organ injury and systemic inflammatory responses—as correlates of disease severity in SFTS [6–8,26]. Compared with these routinely used admission markers that predominantly reflect downstream damage, sCD163 captures upstream macrophage activation and therefore offers complementary prognostic information. sCD163 has shown prognostic utility in sepsis, acute liver failure and other hyperinflammatory states [27–30]. However, whether admission sCD163 improves early risk stratification in SFTS by outperforming routine indices has not been established. In this multicenter study, admission sCD163 was independently associated with in-hospital mortality and achieved a discriminatory cutoff (~1.17 µg/mL) that outperformed single routine indices. Importantly, combining sCD163 with BUN yielded the highest prognostic performance, supporting a risk assessment that integrates upstream immune activation with organ injury markers at admission.

Building on this, we derived a three-tier admission score (sCD163 + BUN) that delivers stepwise risk stratification, facilitating rapid triage at admission, in the emergency department or fever clinic. The rationale for this graduated design rests on the biological complementarity of these two markers. sCD163 captures upstream monocyte/macrophage activation and systemic immune dysregulation, while BUN reflects downstream renal strain and organ injury. Their additive prognostic contributions were confirmed in multivariable modeling. A three-tier rather than binary design was chosen over a binary classification to preserve the intermediate-risk group (Score 1), which represents a clinically distinct cohort warranting closer observation without immediate intensive intervention. This graduated framework directly maps to actionable clinical decisions: Score 0 supports routine monitoring, Score 1 warrants intensified observation, and Score 2 should prompt early escalation of supportive care. Subgroup analyses by age, sex, and corticosteroid use were limited by small sample sizes and did not yield statistically robust results. In exploratory analyses performed after risk stratification, corticosteroid use was associated with lower survival within each risk tier. However, these observations should be interpreted with caution, as they are likely affected by confounding by indication and time-related biases, and the analyses are underpowered for valid inference. In clinical practice, corticosteroids are typically reserved for hyperinflammation, refractory shock, relative adrenal insufficiency, or life-threatening immune-mediated complications. The limited number of steroid-treated patients precluded further analyses stratified by timing, dose, or duration. Larger, prospective, risk-stratified studies are needed to clarify whether specific subgroups may benefit from corticosteroid therapy.

This study has several limitations. First, the cohort comprised only hospitalized patients with SFTS, without outpatients, which may bias the sample toward more severe cases. In many settings, non-hospitalized SFTS presents as clinically mild with very low observed mortality, and thus the current findings are more applicable to admission triage in hospitalized patients. Second, sCD163 was obtained once at admission, by design, to support fast risk stratification. This pragmatic choice limits inference about disease trajectories, underscoring the need for multicenter, serial-sampling cohorts to characterize dynamics and optimize thresholds. Third, the number of outcome events was modest (~18 deaths), constraining the events-per-variable and therefore the complexity of multivariable modeling. Fourth, the external validation cohort and several prespecified subgroups had limited sample sizes, yielding wide confidence intervals and reducing precision; accordingly, some subgroup inferences are unstable and need further study.

## Conclusion

This multicenter study links upstream mechanistic signals to clinical triage in SFTS: single-cell profiling implicates CD163 ⁺ intermediate monocytes with strong antiviral signature and reduced THBS signaling in adverse outcomes, which were orthogonally validated by bulk transcriptomics and flow cytometry. Translating these findings to the bedside, admission sCD163 provides an independent, readily measurable predictor of in-hospital mortality in SFTS. When paired with BUN, it yields a simple three-tier admission score that discriminated risk and was reproduced in an external cohort. Subgroup findings were exploratory and should be validated in larger prospective cohorts. In exploratory, risk-stratified analyses, corticosteroid use was associated with lower survival. However, this observation was driven by confounding and must not be interpreted as a causal adverse effect of the treatment. Prospective, risk-stratified studies are needed to clarify whether any subgroup benefits from corticosteroids. Overall, the sCD163 + BUN tool translates mechanistic findings into a practical bedside tool for early identification of high-risk patients and timely escalation of supportive care.

## Supporting information

S1 Table Differentially expressed genes between non-survivors and survivors in CD163 ⁺ intermediate monocytes. (XLSX)

S2 Table Differentially expressed genes between non-survivors and survivors in CD163 ⁺ classical monocytes. (XLSX)

S3 Table GO enrichment result of highly expressed genes in CD163 ⁺ intermediate monocytes. (XLSX)

S4 Table GO enrichment result of highly expressed genes in CD163 ⁺ classical monocytes. (XLSX)

S5 Table Differentially expressed genes between CD163⁺ and CD163 ⁻ monocytes. (XLSX)

S6 Table GO enrichment result of highly expressed genes in CD163 ⁺ monocytes. (XLSX)

S1 Fig Gene Ontology (GO) enrichment of differentially expressed genes (DEGs) in CD163 ⁺ monocytes compared with CD163 ⁻ monocytes. (TIF)

S2 Fig Comparison of CD163 expression between healthy controls (HCs) and SFTS patients in bulk RNA-seq data. (TIF)

S3 Fig Restricted cubic spline (RCS) model showing the relationship between sCD163 levels and 30-day mortality risk in SFTS patients. (TIF)

S4 Fig Subgroup analyses of the three-tier sCD163 + BUN risk score in SFTS patients. Kaplan Meier survival curves of 30-day mortality according to the three-tier admission risk score combining sCD163 and BUN (Score 0: both normal, sCD163 ≤ 1.17 µg/mL and BUN ≤ 7.1 mmol/L; Score 1: one abnormal; Score 2: both abnormal) across subgroups stratified by age (≥70 or <70 years), sex (male or female), and corticosteroid use (users or non-users). “Number at risk” is shown below each plot.
(TIF)
