# Supplementary figures and images for: CD163⁺ monocytes and soluble CD163 as prognostic indicators in severe fever with thrombocytopenia syndrome: An integrative analysis
Source: PLoS Negl Trop Dis. 2026 Jun 3;20(6):e0014416. doi: 10.1371/journal.pntd.0014416 (PMC13252838; doi:10.1371/journal.pntd.0014416)

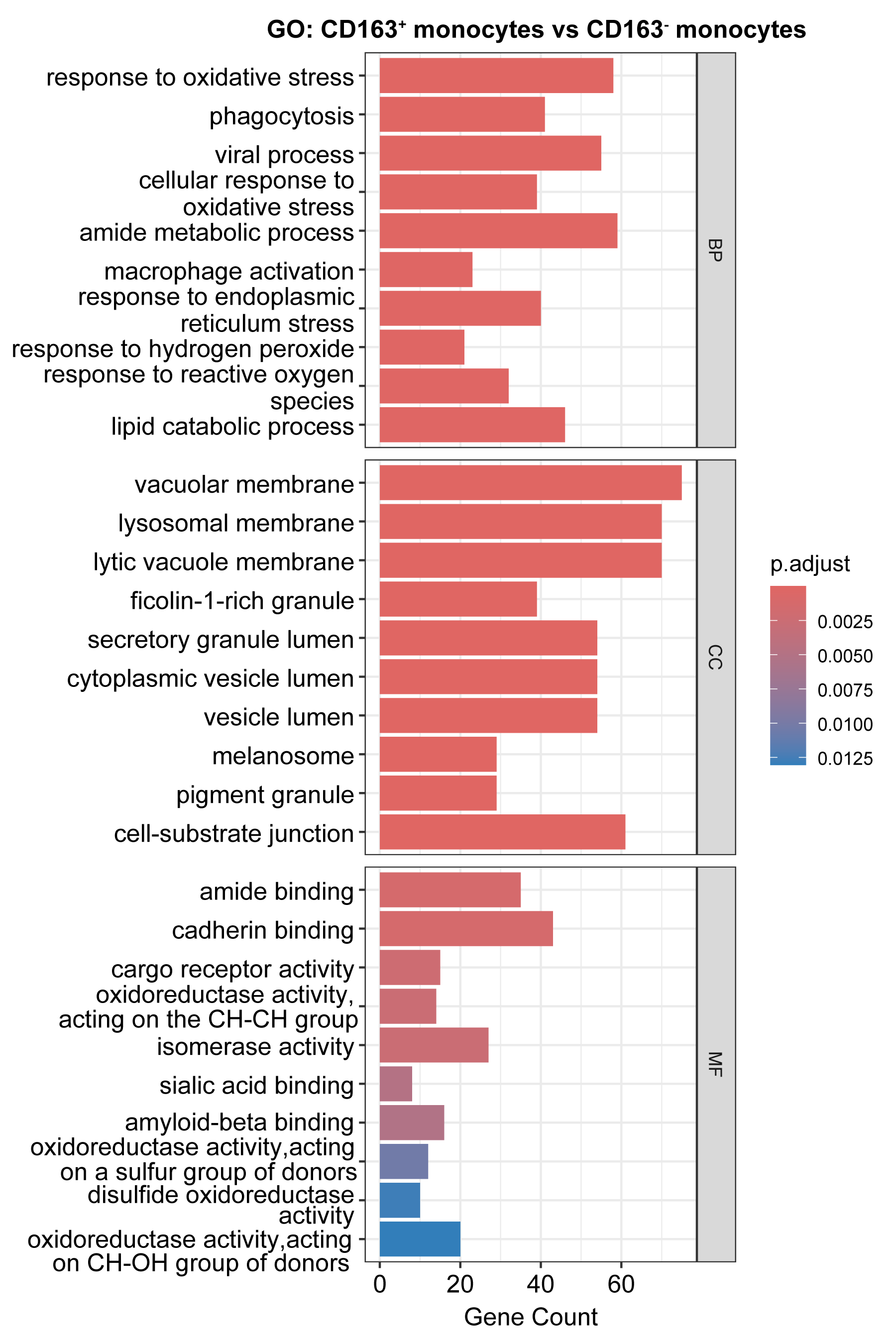

Supplement: S1 Fig — (TIF) [file pntd.0014416.s007.tif]

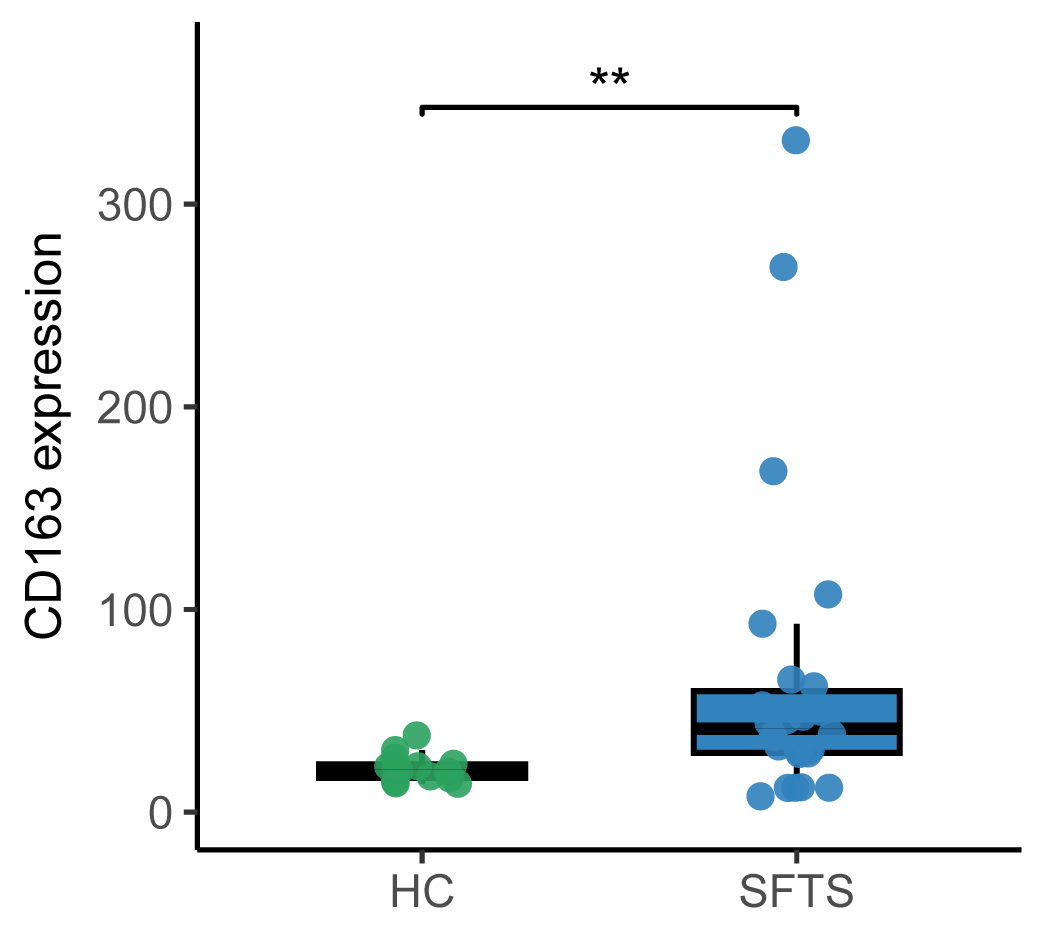

Supplement: S2 Fig — (TIF) [file pntd.0014416.s008.tif]

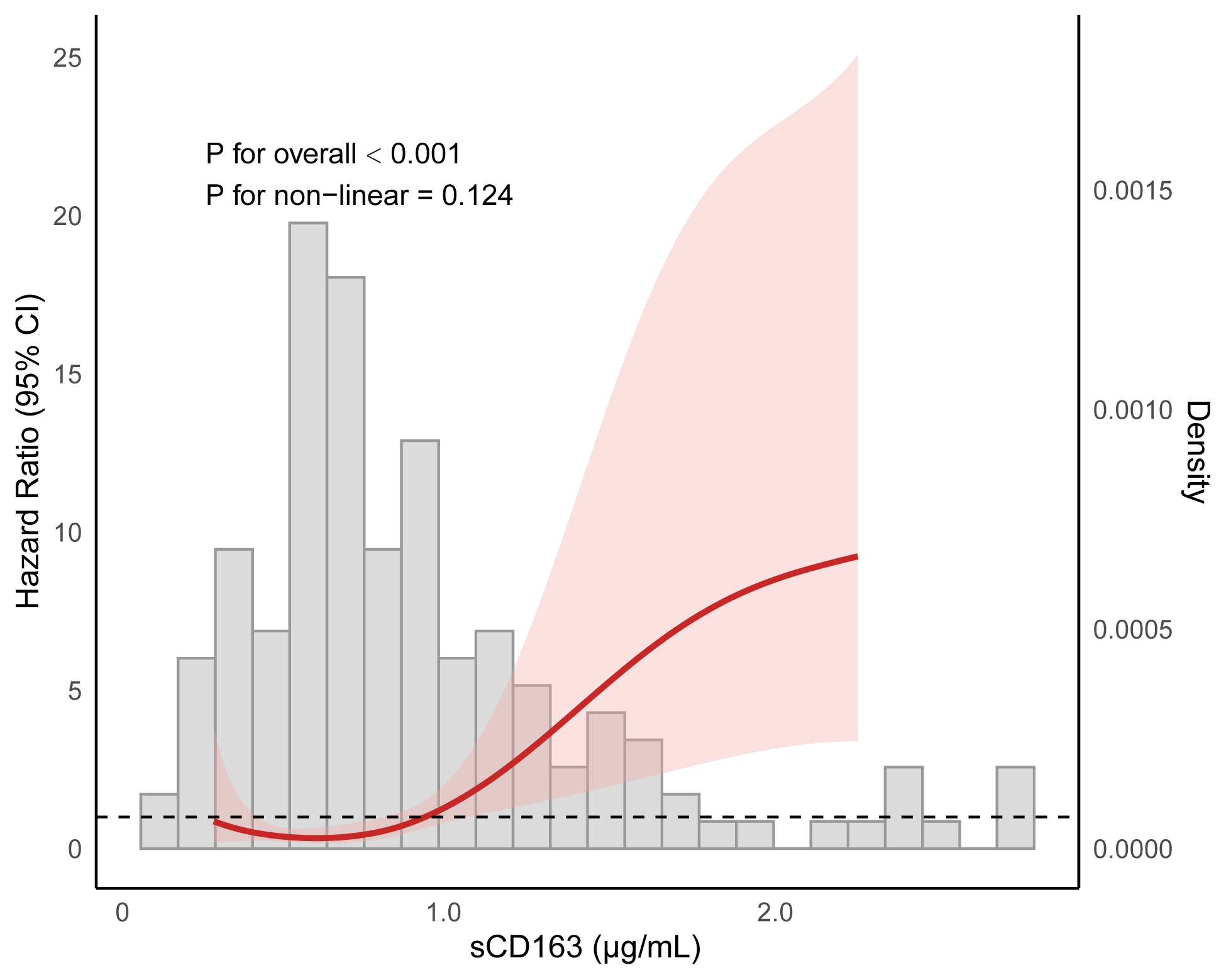

Supplement: S3 Fig — (TIF) [file pntd.0014416.s009.tif]

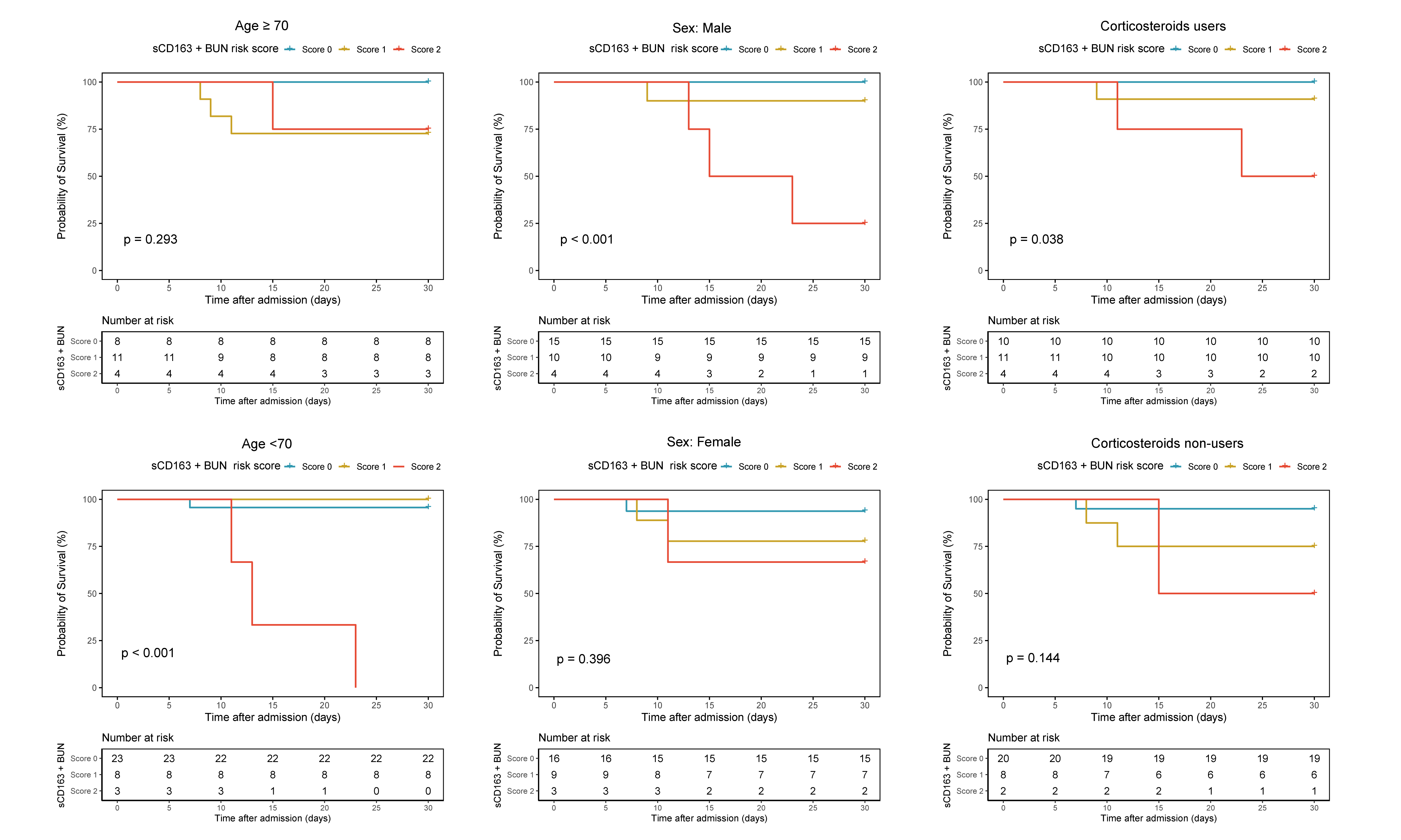

Supplement: S4 Fig — Kaplan Meier survival curves of 30-day mortality according to the three-tier admission risk score combining sCD163 and BUN (Score 0: both normal, sCD163 ≤ 1.17 µg/mL and BUN ≤ 7.1 mmol/L; Score 1: one abnormal; Score 2: both abnormal) across subgroups stratified by age (≥70 or <70 years), sex (male or female), and corticosteroid use (users or non-users). “Number at risk” is shown below each plot. (TIF) [file pntd.0014416.s010.tif]
