# Supplementary material for: CD163⁺ monocytes and soluble CD163 as prognostic indicators in severe fever with thrombocytopenia syndrome: An integrative analysis
Source: PLoS Negl Trop Dis. 2026 Jun 3;20(6):e0014416. doi: 10.1371/journal.pntd.0014416 (PMC13252838; doi:10.1371/journal.pntd.0014416)
Supplement: Attachment — Submitted filename: Response to Reviewers.docx [file pntd.0014416.s012.docx]

Reviewer #1:

Comment 1: The methodology of this paper is logical and nicely performed. The authors also pointed out that the sample size is one of the limitations of this study, which is acceptable.

**Response:** We sincerely thank the reviewer for the positive evaluation of our methodology and the overall study design. We appreciate your understanding regarding the sample size limitations, and we hope to further validate these findings in larger, multi-center prospective cohorts in the future.

Comment 2: There is no major issue with the presentation of results in this manuscript. One suggestion is to clearly define the differences of CD163+/- and classical/intermediate populations in the first part (scRNAseq) of the result section.

**Response:** We thank the reviewer for this constructive suggestion. We agree that clearly defining these monocyte subpopulations at the beginning of the scRNA-seq Results section improves clarity, and we have revised the manuscript accordingly.

Monocyte subsets were annotated based on their transcriptomic profiles, guided by canonical marker expression and consistent with established monocyte classification frameworks in human peripheral blood[1]. Clusters with dominant CD14 expression and low CD16 were assigned as classical monocytes, whereas clusters co-expressing CD14 and CD16 were assigned as intermediate monocytes (Fig. 1C-D). Each subset was further divided by CD163 expression into CD163⁺ and CD163⁻ populations, yielding four phenotypic groups: CD163⁺ classical (clusters 1, 3), CD163⁺ intermediate (clusters 0, 4), CD163⁻ classical (clusters 2, 5), and CD163⁻ intermediate (clusters 6, 7) (Fig. 1C-D).

The corresponding text has been revised in the Results section (Page 9–10, Lines 199–208).

Comment3: The conclusions of this study are mostly straight forward and clear. However, the reason to combine BUN reading with sCD163 for tier analysis seems weak and didn't fully explain.

**Response:** We thank the reviewer for this important comment. We have clarified the rationale for combining sCD163 with BUN in a three-tier risk scoring system.

1、**Additive prognostic value:** Both markers independently predict 30-day mortality, as confirmed by multivariable Cox regression (Table 2). The BUN threshold (7.1 mmol/L) was selected based on prior studies[2] and clinical experience, consistent with established prognostic indices. ROC analyses showed that combining sCD163 with BUN increased predictive accuracy (AUC 0.87) beyond individual markers as sCD163 (AUC 0.80) or sCD163 + APTT (AUC 0.83), supporting an additive effect rather than a simple threshold.

2**、Practical clinical utility**：The three-tier system preserves an intermediate-risk group (Score 1) that may benefit most from early intensified monitoring, providing clearer separation of survival trajectories than binary classification (Fig. 5A–F). Clinically, the tiers correspond to actionable strategies: Score 0, routine monitoring; Score 1, closer observation; Score 2, early intensive intervention.

The Results section now explicitly describes the prognostic contribution and additive effect of each marker (Page 17, Lines 360–364), while the Discussion highlights the biological complementarity of sCD163 (upstream immune activation) and BUN (downstream organ injury).

Comment3: Also, the relationship between CD8+ T cells and CD163+ cells (Line 447-448) seems out from nowhere, it also need more data (in the result) or references (in the introduction/discussion) to support it.

**Response:** We thank the reviewer for this valuable suggestion and apologize for the abrupt introduction of this relationship without adequate support. We have addressed this as follows:

We have now added the corresponding CellChat results to the Results section. Specifically, we clarified that although CD163⁺ intermediate monocytes were among the top signal receivers in both groups, CD8⁺ T cells showed the highest overall incoming signaling strength in non-survivors. In addition, CD163⁺ monocytes displayed strong outgoing communication toward CD8⁺ T cells in non-survivors (Fig. 3A). These additions provide direct data support for the CD163⁺ monocyte–CD8⁺ T cell relationship described in the Discussion and avoid introducing this association without prior evidence. The revised text has been added to the Results section (Page 12, Lines 265–268).

Comment4: The major issue of this manuscript is the language. The writing language seems oversimplified yet hard for the readers to understand. There are many specific and/or clinical words need more description and rephrasing. (such as KM finding in Line 480). Also, the manuscript needs a thorough proof reading to unify the ways authors presenting exam outcome units and p values, etc. The subtitles in each sections (especially the result section) are not informative and not proper. One may suggest that a complete list of abbreviations could be better than multiple ones in each figure and table legend.

Response：We sincerely thank the reviewer for pointing out these important issues regarding the presentation and readability of our manuscript. We have carefully addressed each of your concerns:

1. Language and Professional Terminology: We have conducted thorough proofreading and revision of the entire manuscript to improve the academic tone, flow, and clarity. We specifically re-evaluated all clinical and statistical jargon to ensure they are adequately described rather than oversimplified.

2. Standardization of Formatting (Units and P-values): We have carefully audited the manuscript to unify the formatting of statistical parameters and clinical units. Throughout the text and tables, all P values are now uniformly presented (e.g., *P* < 0.05, *P* = 0.01) using an italicized uppercase *P* with consistent spacing. Clinical units have also been standardized (e.g., consistently using a space between the number and unit, such as 1.17 µg/mL).

3. Informative Subtitles: We agree that the previous subtitles in the Results section were not sufficiently informative. We have completely rewritten the subheadings to be declarative, summarizing the key biological or clinical takeaway of each section rather than simply stating the topic. (e.g., changing "Orthogonal validation in independent modalities" to "Bulk transcriptomics and flow cytometry orthogonally validate CD163⁺ monocyte expansion and functional dysregulation").

4. Abbreviations: Following the reviewer’s helpful suggestion, we have compiled a comprehensive list of abbreviations and added it after the Conclusion section in the revised manuscript. Accordingly, we have streamlined the figure and table legends by removing repetitive definitions of common abbreviations, while retaining only those necessary for standalone understanding of each specific figure or table. These revisions make the legends cleaner and more concise.

Comment5: Overall, the authors, by re-analyzing exist scRNAseq data, have shown a clear clinical method to evaluate the disease outcome of SFTS, which may help early diagnosis of possible severe symptoms. The design of this study and the methods to analyze the observations are logic and proper. The data are also well presented. However, the writing language is not ideal, many points the authors made need more explanations. A thorough proof reading and rephrasing may also improve the reading experience of readers who are interested in this manuscript.

Response：We sincerely appreciate your encouraging summary and positive evaluation of our study design, methodology, and data presentation. We take your feedback regarding the writing quality and the need for deeper explanations very seriously. As detailed in our responses to Comments 3 and 4 above, we have thoroughly proofread and polished the entire manuscript, refined our clinical terminology, and expanded our explanations of the key rationales to ensure a clearer and more informative reading experience. We hope the revised manuscript now meets your expectations and the high standards of PLOS Neglected Tropical Diseases.

Reviewer #2:

Major Comments：

Comment1: Invalid subgroup analyses due to small sample size (Figure 6 & Figure S4):
The manuscript claims the risk score performs well across various subgroups (age, sex, corticosteroid use). However, a close inspection of the "Number at risk" tables (especially in Figure S4) reveals that the sample sizes are insufficient for valid statistical inference.
In Figure S4, the high-risk "Score 2" group contains only n=3 patients in the "Age < 70" stratum, n=3 in the "Female" stratum, and n=2 in the "Corticosteroids non-users" stratum. Calculating Hazard Ratios (HR) or P-values on groups of 2 or 3 individuals is statistically meaningless and prone to extreme noise.
The authors must remove the strong claims regarding the score's consistent performance in these specific subgroups from the Abstract and Discussion. I strongly recommend removing Figure S4 entirely or moving it to a supplementary file with a prominent disclaimer that the study is underpowered for such stratification.

**Response****:** We thank the reviewer for carefully reviewing our data and providing a detailed statistical critique. We completely agree that calculating HR or P-values in strata with extremely small sample sizes (e.g., n=2 or 3 in the Score 2 subgroups) is statistically unreliable. In response, we have revised the manuscript as follows:

**1. Removal of Unsupported Claims:** All statements in the Abstract, Results and Discussion asserting the risk score’s “consistent performance” across these subgroups have been removed. We now explicitly state that the study is underpowered for such stratification and that validation in larger prospective cohorts is needed (Page 2, Line 42–43, Page 24, Line 537–538).

**2. Retention with a Prominent Disclaimer:** In the interest of transparency, Figure S4 has been retained in the supplementary materials. The limitation disclaimer of Figure S4 has been added in Result (Page 18–19, Line 397–400) and Discussion (Page 23, Line 505–506).

Comment2: Confounding in corticosteroid interpretation (Figure 6B):
The Abstract and Results imply a link between corticosteroid use and lower survival ("...link between corticosteroid use and lower survival...").
As shown in Figure 6B, the comparison in the high-risk group involves extremely small numbers (n=7 vs n=8). More importantly, this analysis does not account for confounding by indication—patients receiving corticosteroids were likely more critically ill (e.g., with encephalopathy or shock) than those who did not. Without propensity score matching (which is not feasible here due to sample size), implying a negative effect of the drug is scientifically unsafe.
The authors must rewrite these sections. The association should be framed strictly as an observation likely driven by baseline disease severity differences. The phrasing in the Abstract must be revised to avoid suggesting a causal link.

Response：We sincerely appreciate the reviewer’s comment regarding the potential confounding in the observed association between corticosteroid use and survival. We fully agree that, given the small sample sizes in the high-risk group and the likely differences in baseline disease severity, these analyses cannot support causal inference. To address this, we have revised the text as follows:

1. **Abstract**: We had replaced the original statement in the Abstract with a cautious phrasing clarifying that the observation is exploratory and likely confounded (Page 2, Line 41–43).

2. **Result/Discussion:** The description of Figure 6B has been clarified to indicate that the observed survival differences by corticosteroid use are exploratory and likely influenced by baseline disease severity and other confounders (Page 18–19, Line 397–400; Page 23–24, Line 506–515, 538–541).

Comment 3: Statistical independence in scRNA-seq analysis (Figure 1E & 1F):
The manuscript reports highly significant differences (***, P < 0.001) in monocyte subset proportions between survivors (n=11) and non-survivors (n=4).
Please clarify the statistical method used. If the test treated individual cells as replicates, this constitutes pseudo-replication, artificially inflating the P-value.
Comparisons of cell proportions must be performed using the patient (n) as the biological replicate (e.g., Mann-Whitney U test on percentages per patient). If the P-value changes using the correct method, the significance level in Figure 1 should be updated.

Response: We thank the reviewer for this important comment. We agree that comparisons of monocyte subset proportions in scRNA-seq data should use individual patients, rather than individual cells, as biological replicates to avoid pseudo-replication. We therefore reanalyzed the proportions of monocyte subsets by calculating the percentage of each subset for each patient and comparing groups using the Mann–Whitney U test.

In this patient-level reanalysis, SFTS patients still showed a significant expansion of total CD163⁺ monocytes compared with healthy controls (P = 0.024), particularly CD163⁺ intermediate monocytes (P = 0.003; revised Fig. 1E). This supports the enrichment of the CD163⁺ monocyte compartment in SFTS. However, when the same patient-level approach was applied to compare non-survivors and survivors, the differences in CD163⁺ intermediate and CD163⁺ classical monocytes were no longer statistically significant. Accordingly, we have updated Figure 1E and 1F and revised the corresponding Results text to avoid overstating the survivor versus non-survivor comparison.

Notably, CD163⁺ intermediate monocytes remained numerically higher in non-survivors than in survivors (median 41.7% vs. 37.1%), whereas CD163⁺ classical monocytes were numerically lower in non-survivors (median 24.9% vs. 36.9%). Given the small number of non-survivors in the scRNA-seq cohort (n = 4), this analysis was underpowered for robust statistical inference. We now present the survivor versus non-survivor findings as descriptive observations rather than statistically significant differences.

Comment 4: Uncertainty in validation cohort (Figure 5F):
In the external validation cohort, the high-risk "Score 2" group starts with only n=7 patients. By Day 30, only ~3 patients remain at risk.
Please add 95% Confidence Interval (CI) shading to the Kaplan-Meier curves in Figure 5 (C, E, F). This will visually represent the uncertainty and allow readers to better assess the reliability of the separation between risk groups.

Response: We thank the reviewer for this valuable suggestion. In the revised manuscript, we have added 95% confidence interval (CI) shading to the Kaplan–Meier curves in Figure 5C, 5E, and 5F to visually convey the uncertainty associated with small sample sizes, particularly in the high-risk (Score 2) group of the external validation cohort.

Minor Comments：

1. Linearity of risk (Figure S3): The restricted cubic spline analysis (Fig S3) is a valuable addition, showing a linear relationship (P for non-linear = 0.124). It would be helpful to explicitly state in the Results that "mortality risk increases linearly with sCD163 levels," which supports its use as a continuous marker.

Response: We thank the reviewer for highlighting the importance of explicitly stating the linearity of risk. We have added the following sentence to the Results (Page 17, Line 368–370).

2. Figure 2 and Table S1 show high expression of inflammatory chemokines (CXCL10, ISG15) in CD163+ monocytes, while Figure 4B shows significantly low HLA-DR expression (immunoparalysis). Please briefly discuss this "hyper-inflammatory yet immunoparalyzed" phenotype in the Discussion, as it is a complex feature of severe viral sepsis.

Response: We thank the reviewer for this helpful suggestion. We agree that the coexistence of inflammatory activation and reduced HLA-DR expression in CD163⁺ monocytes is an important feature that warrants further discussion. We have added a new paragraph in the Discussion (Page 22, Lines 469–479) to address this “hyper-inflammatory yet immunoparalyzed” phenotype. In this paragraph, we now state that CD163⁺ monocytes showed increased expression of inflammatory and interferon-stimulated mediators, including CXCL10 and ISG15, together with reduced HLA-DR surface expression, suggesting impaired antigen-presenting capacity. We further interpret this finding in the context of sepsis-induced monocyte reprogramming, in which pro-inflammatory and immunosuppressive features may coexist during a dysregulated host response to infection. We also cite previous studies showing that reduced monocyte HLA-DR expression is a hallmark of monocyte anergy or immunoparalysis and is associated with impaired antigen presentation, secondary infections, and poor outcomes in sepsis.

3. In the Abstract and Introduction, please explicitly state that the scRNA-seq data is a re-analysis of a public dataset (GSE175499) to clearly distinguish it from the newly recruited clinical cohorts.

Response: We sincerely appreciate the reviewer’s comment. In response, we have changed the abstract as “Public single-cell RNA sequencing (scRNA-seq) data (GSE175499) from 15 SFTS patients (4 non-survivors, 11 survivors) and 4 healthy controls were reanalyzed to map cell–cell communication and identify monocyte subsets and pathways” (Page 2, Line 25).

In the introduction, we added “Here, we pursue a translational strategy spanning discovery to bedside. We integrated single-cell RNA sequencing (scRNA-seq) from the public dataset (GSE175499)” (Page 4, Line 91).

4. In Table 1, some P-values are listed as ">0.999" or ">0.900". Please use standard formatting (e.g., P > 0.99 or P = 0.92).

Response: We thank the reviewer for pointing out the inconsistent formatting of P values in Table 1. We have carefully checked Table 1 and standardized the presentation of all P values. Values previously shown as “>0.999” or “>0.900” have been revised to standard notation.

References

1.	Li H, Li X, Lv S, Peng X, Cui N, Yang T, et al. Single-cell landscape of peripheral immune responses to fatal SFTS. Cell Rep. 2021;37: 110039. doi:10.1016/j.celrep.2021.110039

2.	Kirtane AJ, Leder DM, Waikar SS, Chertow GM, Ray KK, Pinto DS, et al. Serum blood urea nitrogen as an independent marker of subsequent mortality among patients with acute coronary syndromes and normal to mildly reduced glomerular filtration rates. J Am Coll Cardiol. 2005;45: 1781–1786. doi:10.1016/j.jacc.2005.02.068
