# Supplementary material for: CD163⁺ monocytes and soluble CD163 as prognostic indicators in severe fever with thrombocytopenia syndrome: An integrative analysis
Source: PLoS Negl Trop Dis. 2026 Jun 3;20(6):e0014416. doi: 10.1371/journal.pntd.0014416 (PMC13252838; doi:10.1371/journal.pntd.0014416)
Supplement: Peer review history

# Peer review: Decision Letter 0

**DOI:** 10.1371/journal.pntd.0014416.r001

**Authors:** Richard Bowen

12 Apr 2026

PNTD-D-25-02112

CD163+ monocytes and soluble CD163 as prognostic indicators in severe fever with thrombocytopenia syndrome: An integrative analysis

PLOS Neglected Tropical Diseases

Dear Dr. Wu,

Thank you for submitting your manuscript to PLOS Neglected Tropical Diseases. After careful consideration, we feel that it has merit but does not fully meet PLOS Neglected Tropical Diseases's publication criteria as it currently stands. Therefore, we invite you to submit a revised version of the manuscript that addresses the points raised during the review process.

Please submit your revised manuscript within by Jun 11 2026 11:59PM. If you will need more time than this to complete your revisions, please reply to this message or contact the journal office at plosntds@plos.org. When you're ready to submit your revision, log on to https://www.editorialmanager.com/pntd/ and select the 'Submissions Needing Revision' folder to locate your manuscript file.

Please include the following items when submitting your revised manuscript:

* A letter that responds to each point raised by the editor and reviewer(s). You should upload this letter as a separate file labeled 'Response to Reviewers'. This file does not need to include responses to any formatting updates and technical items listed in the 'Journal Requirements' section below.

* A marked-up copy of your manuscript that highlights changes made to the original version. You should upload this as a separate file labeled 'Revised Manuscript with Track Changes'.

* An unmarked version of your revised paper without tracked changes. You should upload this as a separate file labeled 'Manuscript'.

* A Data availability statement and inclusion of the data in a permanent database (e.g. Zenodo)

If you would like to make changes to your financial disclosure, competing interests statement, or data availability statement, please make these updates within the submission form at the time of resubmission. Guidelines for resubmitting your figure files are available below the reviewer comments at the end of this letter.

We look forward to receiving your revised manuscript.

Kind regards,

Richard A. Bowen, DVM PhD

Academic Editor

PLOS Neglected Tropical Diseases

Nikos Vasilakis

Section Editor

PLOS Neglected Tropical Diseases

Shaden Kamhawi

co-Editor-in-Chief

PLOS Neglected Tropical Diseases

orcid.org/0000-0003-4304-636XX

Paul Brindley

co-Editor-in-Chief

PLOS Neglected Tropical Diseases

orcid.org/0000-0003-1765-0002

**Additional Editor Comments:**

Your manuscript has been reviewed by two experts and each has provided feedback on deficiencies or points of clarification the consider should be addressed. Please evaluate these reviewer comments, edit your your manuscript as you feel appropriate and resubmit along with a Response to Reviewers document detailing the changes you have made or justification for not editing your manuscript. Thank you and apologies for the length of time it took to get these reviews.

Include a data availability statement and a link to a permanent data repository database (e.g., Zenodo)

**Journal Requirements:**

If the reviewer comments include a recommendation to cite specific previously published works, please review and evaluate these publications to determine whether they are relevant and should be cited. There is no requirement to cite these works unless the editor has indicated otherwise.

1) Please ensure that the CRediT author contributions listed for every co-author are completed accurately and in full.

At this stage, the following Authors/Authors require contributions: Jie Wang, Haolin Song, Yi Zhang, Ziling Cheng, Lingtong Huang, Bei Jia, Guangqi Zhu, Lifen Hu, Jihua Xue, Jie Li, Wei Wu, and Qi Xia. Please ensure that the full contributions of each author are acknowledged in the "Add/Edit/Remove Authors" section of our submission form.

The list of CRediT author contributions may be found here: https://journals.plos.org/plosntds/s/authorship#loc-author-contributions

2) Please amend your detailed Financial Disclosure statement. This is published with the article. It must therefore be completed in full sentences and contain the exact wording you wish to be published.

1) State what role the funders took in the study. If the funders had no role in your study, please state: "The funders had no role in study design, data collection and analysis, decision to publish, or preparation of the manuscript."

2) If any authors received a salary from any of your funders, please state which authors and which funders..

If you did not receive any funding for this study, please simply state: u201cThe authors received no specific funding for this work.u201d

**Reviewers' Comments:**

Reviewer's Responses to Questions

**Key Review Criteria Required for Acceptance?**

As you describe the new analyses required for acceptance, please consider the following:

**Methods**

-Are the objectives of the study clearly articulated with a clear testable hypothesis stated?

-Is the study design appropriate to address the stated objectives?

-Is the population clearly described and appropriate for the hypothesis being tested?

-Is the sample size sufficient to ensure adequate power to address the hypothesis being tested?

-Were correct statistical analysis used to support conclusions?

-Are there concerns about ethical or regulatory requirements being met?

Reviewer #1: The methodology of this paper is logical and nicely performed. The authors also pointed out that the sample size is one of the limitations of this study, which is acceptable.

Reviewer #2: Provided in Summary and General Comments.

**********

**Results**

-Does the analysis presented match the analysis plan?

-Are the results clearly and completely presented?

-Are the figures (Tables, Images) of sufficient quality for clarity?

Reviewer #1: There is no major issue with the presentation of results in this manuscript. One suggestion is to clearly define the differences of CD163+/- and classical/intermediate populations in the first part (scRNAseq) of the result section.

Reviewer #2: Provided in Summary and General Comments.

**********

**Conclusions**

-Are the conclusions supported by the data presented?

-Are the limitations of analysis clearly described?

-Do the authors discuss how these data can be helpful to advance our understanding of the topic under study?

-Is public health relevance addressed?

Reviewer #1: The conclusions of this study are mostly straight forward and clear. However, the reason to combine BUN reading with sCD163 for tier analysis seems weak and didn't fully explained. Also, the relationship between CD8+ T cells and CD163+ cells (Line 447-448) seems out from nowhere, it also need more data (in the result) or references (in the introduction/discussion) to support it.

Reviewer #2: Provided in Summary and General Comments.

**********

**Editorial and Data Presentation Modifications?**

Use this section for editorial suggestions as well as relatively minor modifications of existing data that would enhance clarity. If the only modifications needed are minor and/or editorial, you may wish to recommend “Minor Revision” or “Accept”.

Reviewer #1: The major issue of this manuscript is the language. The writing language seems oversimplified yet hard for the readers to understand. There are many specific and/or clinical words need more description and rephrasing. (such as KM finding in Line 480). Also, the manuscript needs a thorough proof reading to unify the ways authors presenting exam outcome units and p values, etc. The subtitles in each sections (especially the result section) are not informative and not proper. One may suggest that a complete list of abbreviations could be better than multiple ones in each figure and table legend.

Reviewer #2: Provided in Summary and General Comments.

**********

**Summary and General Comments**

Use this section to provide overall comments, discuss strengths/weaknesses of the study, novelty, significance, general execution and scholarship. You may also include additional comments for the author, including concerns about dual publication, research ethics, or publication ethics. If requesting major revision, please articulate the new experiments that are needed.

Reviewer #1: Overall, the authors, by re-analyzing exist scRNAseq data, have shown a clear clinical method to evaluate the disease outcome of SFTS , which may help early diagnosis of possible severe symptoms. The design of this study and the methods to analyze the observations are logic and proper. The data are also well presented. However, the writing language is not ideal, many points the authors made need more explanations. A thorough proof reading and rephrasing may also improve the reading experience of readers who are interested in this manuscript.

Reviewer #2: Summary

This study presents an integrative analysis combining scRNA-seq re-analysis and multi-center clinical cohorts to identify CD163+ monocytes and serum sCD163 as prognostic markers for SFTS. The authors further propose a pragmatic "sCD163 + BUN" risk score for early risk stratification.

The study has significant strengths, particularly its "mechanism-to-clinic" design. The characterization of the CD163+ monocyte subset is robust, supported by clear scRNA-seq data (Fig 1, Table S1-S2) showing a distinct antiviral/inflammatory signature, and orthogonally validated by flow cytometry (Fig 4). The restricted cubic spline analysis (Fig S3) provides strong evidence for a linear dose-response relationship between sCD163 and mortality.

However, I have substantial concerns regarding the statistical robustness of the clinical validation, specifically the subgroup analyses and the interpretation of corticosteroid effects. The sample sizes in high-risk strata are too small to support the broad conclusions currently made in the text.

Major Comments

1. Invalid subgroup analyses due to small sample size (Figure 6 & Figure S4):

The manuscript claims the risk score performs well across various subgroups (age, sex, corticosteroid use). However, a close inspection of the "Number at risk" tables (especially in Figure S4) reveals that the sample sizes are insufficient for valid statistical inference.

In Figure S4, the high-risk "Score 2" group contains only n=3 patients in the "Age < 70" stratum, n=3 in the "Female" stratum, and n=2 in the "Corticosteroids non-users" stratum. Calculating Hazard Ratios (HR) or P-values on groups of 2 or 3 individuals is statistically meaningless and prone to extreme noise.

The authors must remove the strong claims regarding the score's consistent performance in these specific subgroups from the Abstract and Discussion. I strongly recommend removing Figure S4 entirely or moving it to a supplementary file with a prominent disclaimer that the study is underpowered for such stratification.

2. Confounding in corticosteroid interpretation (Figure 6B):

The Abstract and Results imply a link between corticosteroid use and lower survival ("...link between corticosteroid use and lower survival...").

As shown in Figure 6B, the comparison in the high-risk group involves extremely small numbers (n=7 vs n=8). More importantly, this analysis does not account for confounding by indication—patients receiving corticosteroids were likely more critically ill (e.g., with encephalopathy or shock) than those who did not. Without propensity score matching (which is not feasible here due to sample size), implying a negative effect of the drug is scientifically unsafe.

The authors must rewrite these sections. The association should be framed strictly as an observation likely driven by baseline disease severity differences. The phrasing in the Abstract must be revised to avoid suggesting a causal link.

3. Statistical independence in scRNA-seq analysis (Figure 1E & 1F):

The manuscript reports highly significant differences (***, P < 0.001) in monocyte subset proportions between survivors (n=11) and non-survivors (n=4).

Please clarify the statistical method used. If the test treated individual cells as replicates, this constitutes pseudo-replication, artificially inflating the P-value.

Comparisons of cell proportions must be performed using the patient (n) as the biological replicate (e.g., Mann-Whitney U test on percentages per patient). If the P-value changes using the correct method, the significance level in Figure 1 should be updated.

4. Uncertainty in validation cohort (Figure 5F):

In the external validation cohort, the high-risk "Score 2" group starts with only n=7 patients. By Day 30, only ~3 patients remain at risk.

Please add 95% Confidence Interval (CI) shading to the Kaplan-Meier curves in Figure 5 (C, E, F). This will visually represent the uncertainty and allow readers to better assess the reliability of the separation between risk groups.

Minor Comments

1. Linearity of risk (Figure S3): The restricted cubic spline analysis (Fig S3) is a valuable addition, showing a linear relationship (P for non-linear = 0.124). It would be helpful to explicitly state in the Results that "mortality risk increases linearly with sCD163 levels," which supports its use as a continuous marker.

2. Figure 2 and Table S1 show high expression of inflammatory chemokines (CXCL10, ISG15) in CD163+ monocytes, while Figure 4B shows significantly low HLA-DR expression (immunoparalysis). Please briefly discuss this "hyper-inflammatory yet immunoparalyzed" phenotype in the Discussion, as it is a complex feature of severe viral sepsis.

3. In the Abstract and Introduction, please explicitly state that the scRNA-seq data is a re-analysis of a public dataset (GSE175499) to clearly distinguish it from the newly recruited clinical cohorts.

4. In Table 1, some P-values are listed as ">0.999" or ">0.900". Please use standard formatting (e.g., P > 0.99 or P = 0.92).

**********

PLOS authors have the option to publish the peer review history of their article (what does this mean?). If published, this will include your full peer review and any attached files.

If you choose “no”, your identity will remain anonymous but your review may still be made public.

**Do you want your identity to be public for this peer review?** For information about this choice, including consent withdrawal, please see our Privacy Policy.

Reviewer #1: No

Reviewer #2: No

**Figure resubmission:**

While revising your submission, we strongly recommend that you use PLOS’s NAAS tool (https://ngplosjournals.pagemajik.ai/artanalysis) to test your figure files. NAAS can convert your figure files to the TIFF file type and meet basic requirements (such as print size, resolution), or provide you with a report on issues that do not meet our requirements and that NAAS cannot fix.

-->After uploading your figures to PLOS’s NAAS tool - https://ngplosjournals.pagemajik.ai/artanalysis, NAAS will process the files provided and display the results in the "Uploaded Files" section of the page as the processing is complete. If the uploaded figures meet our requirements (or NAAS is able to fix the files to meet our requirements), the figure will be marked as "fixed" above. If NAAS is unable to fix the files, a red "failed" label will appear above. When NAAS has confirmed that the figure files meet our requirements, please download the file via the download option, and include these NAAS processed figure files when submitting your revised manuscript.

**Reproducibility:**

To enhance the reproducibility of your results, we recommend that authors of applicable studies deposit laboratory protocols in protocols.io, where a protocol can be assigned its own identifier (DOI) such that it can be cited independently in the future. Additionally, PLOS ONE offers an option to publish peer-reviewed clinical study protocols. Read more information on sharing protocols at https://plos.org/protocols?utm_medium=editorial-email&utm_source=authorletters&utm_campaign=protocols

# Author comment: Author response to Decision Letter 1

**DOI:** 10.1371/journal.pntd.0014416.r002

8 May 2026

Attachment Submitted filename: Response to Reviewers.docx

# Peer review: Decision Letter 1

**DOI:** 10.1371/journal.pntd.0014416.r003

**Authors:** Richard Bowen

27 May 2026

Dear Dr. Wu,

We are pleased to inform you that your manuscript 'CD163+ monocytes and soluble CD163 as prognostic indicators in severe fever with thrombocytopenia syndrome: An integrative analysis' has been provisionally accepted for publication in PLOS Neglected Tropical Diseases.

Before your manuscript can be formally accepted you will need to complete some formatting changes, which you will receive in a follow up email. A member of our team will be in touch with a set of requests.

Please note that your manuscript will not be scheduled for publication until you have made the required changes, so a swift response is appreciated.

IMPORTANT: The editorial review process is now complete. PLOS will only permit corrections to spelling, formatting or significant scientific errors from this point onwards. Requests for major changes, or any which affect the scientific understanding of your work, will cause delays to the publication date of your manuscript.

Should you, your institution's press office or the journal office choose to press release your paper, you will automatically be opted out of early publication. We ask that you notify us now if you or your institution is planning to press release the article. All press must be co-ordinated with PLOS.

Thank you again for supporting Open Access publishing; we are looking forward to publishing your work in PLOS Neglected Tropical Diseases.

Best regards,

Richard A. Bowen, DVM PhD

Academic Editor

PLOS Neglected Tropical Diseases

Nikos Vasilakis

Section Editor

PLOS Neglected Tropical Diseases

Shaden Kamhawi

co-Editor-in-Chief

PLOS Neglected Tropical Diseases

orcid.org/0000-0003-4304-636XX

Paul Brindley

co-Editor-in-Chief

PLOS Neglected Tropical Diseases

orcid.org/0000-0003-1765-0002

***********************************************************

Thank you for the thoughtful response to the first review. One reviewer had some additional minor comments but I have marked this manuscript as accepted.

p.p1 {margin: 0.0px 0.0px 0.0px 0.0px; line-height: 16.0px; font: 14.0px Arial; color: #323333; -webkit-text-stroke: #323333}span.s1 {font-kerning: none

Reviewer's Responses to Questions

**Key Review Criteria Required for Acceptance?**

As you describe the new analyses required for acceptance, please consider the following:

**Methods**

-Are the objectives of the study clearly articulated with a clear testable hypothesis stated?

-Is the study design appropriate to address the stated objectives?

-Is the population clearly described and appropriate for the hypothesis being tested?

-Is the sample size sufficient to ensure adequate power to address the hypothesis being tested?

-Were correct statistical analysis used to support conclusions?

-Are there concerns about ethical or regulatory requirements being met?

Reviewer #1: (No Response)

Reviewer #2: Provided in General Comments.

**********

**Results**

-Does the analysis presented match the analysis plan?

-Are the results clearly and completely presented?

-Are the figures (Tables, Images) of sufficient quality for clarity?

Reviewer #1: (No Response)

Reviewer #2: Yes, it's ok.

**********

**Conclusions**

-Are the conclusions supported by the data presented?

-Are the limitations of analysis clearly described?

-Do the authors discuss how these data can be helpful to advance our understanding of the topic under study?

-Is public health relevance addressed?

Reviewer #1: (No Response)

Reviewer #2: Provided in General Comments.

**********

**Editorial and Data Presentation Modifications?**

Use this section for editorial suggestions as well as relatively minor modifications of existing data that would enhance clarity. If the only modifications needed are minor and/or editorial, you may wish to recommend “Minor Revision” or “Accept”.

Reviewer #1: (No Response)

Reviewer #2: Minor Revision.

**********

**Summary and General Comments**

Use this section to provide overall comments, discuss strengths/weaknesses of the study, novelty, significance, general execution and scholarship. You may also include additional comments for the author, including concerns about dual publication, research ethics, or publication ethics. If requesting major revision, please articulate the new experiments that are needed.

Reviewer #1: The authors have replied to questions and suggestions properly. There is no further recommendation to this manuscript.

Reviewer #2: General assessment

The revised manuscript presents an integrative translational study linking CD163⁺ monocyte biology with admission-time prognostic stratification in severe fever with thrombocytopenia syndrome (SFTS). The authors combine public single-cell RNA-seq re-analysis, bulk transcriptomics, flow cytometric validation, and multicenter clinical cohorts to propose serum sCD163, particularly when combined with BUN, as a prognostic marker for 30-day mortality.

Several issues still require clarification or further tempering before publication. These mainly concern the robustness of the multivariable survival model, the derivation and validation of the proposed risk score, the interpretation of CellChat-based THBS signaling, and the potential for misinterpretation of the corticosteroid-stratified analyses.

Major comments

1. Stability of the multivariable Cox model should be further justified

The discovery cohort includes 150 patients, but only 18 deaths. Despite the authors’ effort to exclude some variables because of multicollinearity, the final multivariable Cox model still appears to include a relatively large number of covariates compared with the number of outcome events. This raises concerns regarding overfitting, coefficient instability, and inflated effect estimates.

This concern is reflected by the wide confidence interval for high sCD163 in the multivariable model. While the association between sCD163 and mortality is biologically plausible and clinically interesting, the statistical robustness of this estimate should be more carefully addressed.

I suggest that the authors either:

(1). further reduce the number of covariates in the multivariable model based on a pre-specified clinical rationale;

(2). provide a penalized regression analysis, such as penalized Cox regression or LASSO Cox, as a sensitivity analysis; or

(3). perform bootstrap internal validation to assess the stability of the selected predictors and estimated hazard ratios.

At minimum, the limitation related to the low number of outcome events and the potential instability of the multivariable model should be stated more explicitly in the Results or Discussion.

2. The derivation of the two-marker three-tier score needs clearer justification

The proposed three-tier score combining sCD163 and BUN is clinically attractive because of its simplicity. However, the current scoring approach appears largely heuristic: one point is assigned for elevated sCD163 and one point for elevated BUN. The rationale for equal weighting is not sufficiently explained.

The authors should clarify why equal weighting was chosen rather than a coefficient-based model. In addition, the manuscript would be strengthened by providing calibration analysis for the proposed score, such as a calibration plot or another suitable assessment of agreement between predicted and observed mortality risk.

If feasible, decision-curve analysis could also help demonstrate whether the sCD163+BUN score provides clinically meaningful net benefit compared with sCD163 alone, BUN alone, or routine clinical markers. This would be particularly relevant because the manuscript emphasizes bedside triage and clinical utility.

3. The mechanistic interpretation of THBS signaling should be further tempered

The CellChat analysis identifies attenuated THBS signaling in non-survivors, with CD163⁺ monocytes as major signal senders. This is an interesting observation. However, the biological interpretation that insufficient THBS-mediated resolution or clearance may contribute to adverse outcomes remains speculative.

Cell–cell communication inference based on transcriptomic data is hypothesis-generating and does not directly demonstrate functional ligand–receptor activity. Since the manuscript does not include protein-level validation of THBS1/TSP1, receptor activation, or perturbation experiments, the causal language around this pathway should be softened.

For example, phrases implying that attenuated THBS signaling “contributes to” poor outcomes should be replaced with more cautious wording, such as “may be associated with,” “raises the hypothesis that,” or “suggests a potential link.” This would better align the interpretation with the level of evidence.

4. Viral load is an important missing clinical variable

SFTSV viral burden is a major determinant of disease severity and mortality in SFTS. The current analysis does not appear to include viral RNA level, Ct value, or another measure of viral load. This limits interpretation of whether sCD163 provides prognostic information independent of viral replication burden.

If viral load data are available, the authors should consider including them in the clinical analysis or at least as a sensitivity analysis. If such data are unavailable, this should be explicitly acknowledged as a limitation.

Minor comments

1. The single-cell non-survivor sample size should be emphasized more clearly

The scRNA-seq dataset includes only four non-survivors. Although this limitation is mentioned, it should be emphasized more clearly when interpreting differential expression and CellChat analyses. These analyses should be framed as exploratory and hypothesis-generating rather than definitive mechanistic evidence.

2. Clarify the generalizability of the sCD163 cutoff

The proposed sCD163 cutoff of 1.17 µg/mL is useful, but its generalizability across different ELISA platforms, laboratories, sample handling procedures, and populations remains uncertain. The authors should mention that assay-specific calibration and external prospective validation are required before clinical implementation.

3. Provide more details on missing data

The manuscript would benefit from clearer reporting of missing data across clinical variables. It is not fully clear whether all 150 patients in the discovery cohort had complete data for sCD163, BUN, and all covariates included in the Cox models. The authors should specify the extent of missingness and how missing values were handled.

4. Clarify whether proportional hazards assumptions were tested

Because Cox regression is central to the clinical analysis, the authors should state whether proportional hazards assumptions were assessed for the main predictors, particularly sCD163 and BUN.

5. Flow cytometry validation supports CD163 expansion but not the full functional phenotype

The flow cytometry panel includes CD14, CD16, CD163, and HLA-DR, which is appropriate for validating CD163⁺ monocyte expansion and reduced HLA-DR expression. However, the claim of a “hyper-inflammatory yet immunoparalyzed” phenotype would be stronger with additional markers, such as PD-L1, CD86, CCR2, or cytokine-related readouts. I do not consider this essential for acceptance, but the authors should slightly temper this interpretation or mention it as a future direction.

Recommendation

I recommend minor revision before acceptance.

The manuscript is scientifically interesting and clinically relevant, and the revised version has improved substantially. The remaining issues do not necessarily require extensive new experiments, but the authors should further clarify the statistical robustness of their prognostic model, better justify the construction of the risk score, temper mechanistic claims based on computational cell–cell communication analysis, and ensure that the corticosteroid-related findings are not interpreted causally. These revisions would improve the rigor and balance of the manuscript.

**********

PLOS authors have the option to publish the peer review history of their article (what does this mean?). If published, this will include your full peer review and any attached files.

If you choose “no”, your identity will remain anonymous but your review may still be made public.

**Do you want your identity to be public for this peer review?** For information about this choice, including consent withdrawal, please see our Privacy Policy.

Reviewer #1: No

Reviewer #2: No

# Editor report: Acceptance letter

**DOI:** 10.1371/journal.pntd.0014416.r004

**Authors:** Richard Bowen

Dear Dr. Wu,

We are delighted to inform you that your manuscript, "CD163+ monocytes and soluble CD163 as prognostic indicators in severe fever with thrombocytopenia syndrome: An integrative analysis," has been formally accepted for publication in PLOS Neglected Tropical Diseases.

We have now passed your article onto the PLOS Production Department who will complete the rest of the publication process. All authors will receive a confirmation email upon publication.

The corresponding author will soon be receiving a typeset proof for review, to ensure errors have not been introduced during production. Please review the PDF proof of your manuscript carefully, as this is the last chance to correct any scientific or type-setting errors. Please note that major changes, or those which affect the scientific understanding of the work, will likely cause delays to the publication date of your manuscript. Note: Proofs for Front Matter articles (Editorial, Viewpoint, Symposium, Review, etc...) are generated on a different schedule and may not be made available as quickly.

Soon after your final files are uploaded, the early version of your manuscript will be published online unless you opted out of this process. The date of the early version will be your article's publication date. The final article will be published to the same URL, and all versions of the paper will be accessible to readers.

For Research Articles, you will receive an invoice from PLOS for your publication fee after your manuscript has reached the completed accept phase. If you receive an email requesting payment before acceptance or for any other service, this may be a phishing scheme. Learn how to identify phishing emails and protect your accounts at https://explore.plos.org/phishing.

Thank you again for supporting open-access publishing; we are looking forward to publishing your work in PLOS Neglected Tropical Diseases.

Best regards,

Shaden Kamhawi

co-Editor-in-Chief

PLOS Neglected Tropical Diseases

Paul Brindley

co-Editor-in-Chief

PLOS Neglected Tropical Diseases
